# Hybrid ionic–electronic semiconductors for interface engineering of ultra-low-dark-current solution-processed SWIR photodetectors

**DOI:** 10.1093/nsr/nwaf531

**Published:** 2025-11-26

**Authors:** Yunhao Cao, Xiye Yang, Yazhong Wang, Jingwen Chen, Haoran Tang, Chunchen Liu, Kai Zhang, Sheng Dong, Yong Cao, Fei Huang

**Affiliations:** State Key Laboratory of Luminescent Materials and Devices, Guangdong Basic Research Center of Excellence for Energy & Information Polymer Materials, Institute of Polymer Optoelectronic Materials and Devices, School of Materials Science and Engineering, South China University of Technology, Guangzhou 510640, China; Lumidar Technology Co., Ltd., Guangzhou 510530, China; State Key Laboratory of Luminescent Materials and Devices, Guangdong Basic Research Center of Excellence for Energy & Information Polymer Materials, Institute of Polymer Optoelectronic Materials and Devices, School of Materials Science and Engineering, South China University of Technology, Guangzhou 510640, China; Lumidar Technology Co., Ltd., Guangzhou 510530, China; State Key Laboratory of Luminescent Materials and Devices, Guangdong Basic Research Center of Excellence for Energy & Information Polymer Materials, Institute of Polymer Optoelectronic Materials and Devices, School of Materials Science and Engineering, South China University of Technology, Guangzhou 510640, China; State Key Laboratory of Luminescent Materials and Devices, Guangdong Basic Research Center of Excellence for Energy & Information Polymer Materials, Institute of Polymer Optoelectronic Materials and Devices, School of Materials Science and Engineering, South China University of Technology, Guangzhou 510640, China; State Key Laboratory of Luminescent Materials and Devices, Guangdong Basic Research Center of Excellence for Energy & Information Polymer Materials, Institute of Polymer Optoelectronic Materials and Devices, School of Materials Science and Engineering, South China University of Technology, Guangzhou 510640, China; State Key Laboratory of Luminescent Materials and Devices, Guangdong Basic Research Center of Excellence for Energy & Information Polymer Materials, Institute of Polymer Optoelectronic Materials and Devices, School of Materials Science and Engineering, South China University of Technology, Guangzhou 510640, China; Lumidar Technology Co., Ltd., Guangzhou 510530, China; State Key Laboratory of Luminescent Materials and Devices, Guangdong Basic Research Center of Excellence for Energy & Information Polymer Materials, Institute of Polymer Optoelectronic Materials and Devices, School of Materials Science and Engineering, South China University of Technology, Guangzhou 510640, China; State Key Laboratory of Luminescent Materials and Devices, Guangdong Basic Research Center of Excellence for Energy & Information Polymer Materials, Institute of Polymer Optoelectronic Materials and Devices, School of Materials Science and Engineering, South China University of Technology, Guangzhou 510640, China

**Keywords:** hybrid ionic–electronic semiconductor, shortwave infrared, solution-processed, photodetector, imager

## Abstract

Shortwave infrared (SWIR) photodetectors are essential for industrial, medical and security applications, but their adoption is limited by reliance on costly epitaxial semiconductors. Solution-processed organic and colloidal quantum dot (CQD) semiconductors offer a promising alternative, although their devices often suffer from high dark-current under reverse bias. We demonstrate an approach to precise work function modulation in SWIR photodetectors using conjugated polyelectrolytes with mixed counterions. This strategy achieves continuous work function tuning and interfacial defect passivation, significantly reducing dark current in solution-processed photodetectors. The optimized CQD photodetectors exhibit a dark-current density two orders of magnitude lower than that of conventional devices at −0.1 V bias (6.7 × 10^−8^ A cm^−2^), achieving a linear dynamic range of 130 dB, specific detectivity of 4.3 × 10^12^ Jones, and cutoff frequency of 107 kHz under 1550 nm illumination. Integrated with a complementary metal-oxide-semiconductor read-out integrated circuit, the resulting SWIR imager demonstrates high-resolution performance (1280 × 1024 pixels), providing a viable alternative to InGaAs-based imagers.

## Teaser

By leveraging hybrid ionicelectronic semiconductors, a universally applicable strategy breaks the performance bottleneck of solution-processed SWIR photodetectors and unlocks high-performance, low-cost SWIR imaging.

## INTRODUCTION

Shortwave infrared (SWIR) photodetection plays an increasingly pivotal role in astronomical observation, machine vision, security surveillance and biomedical diagnostics [1–3]. However, the widespread adoption of conventional indium gallium arsenide (InGaAs) and mercury cadmium telluride (HgCdTe) focal plane array (FPA) sensors is significantly constrained by their high costs and complex fabrication processes [4–6]. In contrast, solution-processed optoelectronic materials, such as organic semiconductors [7–9] and colloidal quantum dots (CQDs) [10–13], represent an easily processed and cost-effective alternative with remarkable progress in SWIR photodetection [14–16]. These materials show the ability to be integrated with complementary metal-oxide-semiconductor (CMOS)-based read-out integrated circuits (ROICs), eliminating the need for pixel-level patterning. Achieving ultralow dark-current density is paramount for integrating these materials with high-sensitivity ROICs, such as high-gain, low full-well capacitive transimpedance amplifier (CTIA) architectures used in low-flux scenarios [17]. Nevertheless, for existing material systems, the dark current tends to rise sharply as the response wavelength increases [18,19]. This is particularly problematic at critical wavelengths, such as the near-infrared II (NIR-II) region, covering wavelengths from 1000 to 1700 nm, which are vital for applications in artificial intelligence (AI), autonomous driving, eye-safe imaging, internet of things and aerospace fields [20–23].

Dark current in photodetectors (PDs) arises predominantly from tunneling injections, interfacial defect states and thermally excited carriers [19,24–26]. To enhance charge separation and extraction, reverse bias is commonly applied to intensify the internal electric field, thereby improving both response speed and responsivity. However, the implementation of reverse bias also introduces significant drawbacks. Specifically, it amplifies charge injection from the electrodes, resulting in increased dark current, which consequently diminishes the specific detectivity (D*) [24]. To mitigate these adverse effects, precise control of the electrode work function (WF) is essential, as it determines the injection barrier at the electrode/semiconductor interface [19,27]. Traditional strategies for modulating WF in semiconductor devices typically focus on altering electrode or interfacial layer materials. However, achieving continuous and precise control over WF without affecting other material properties remains a significant challenge [28–30]. Due to the lack of model electrodes with tunable WF as research platforms, the influence of interfacial states at electrodes on the performance of PDs remains inadequately understood.

Herein, we present a novel strategy to achieve broad and precise control of a single-electrode WF by leveraging conjugated polyelectrolytes (CPEs) with mixed counterions. These CPEs are in fact hybrid ionic–electronic semiconductors (HIESs) [31–34], where the counterions on the side chains can either dope the conjugated backbone to modulate energy levels and carrier transport properties [35], or interact with electrodes to adjust the interfacial WF [36,37]. While previous studies have shown that varying the conjugated backbones and counterion types can alter the WF and carrier properties of CPEs [38,39], these approaches often involve significant chemistry complexity and limited precision. In contrast, our approach demonstrates that by systematically varying the counterion ratio within a unified conjugated backbone, the HIES layer enables precise WF modulation of the silver (Ag) electrode over a broad range of 4.0–5.1 eV, with a tunable resolution of 0.1 eV. Additionally, we verify that introducing the HIES layer between the transition-metal oxide hole transporting layer (HTL) and the metal electrode not only tunes the WF but also passivates interfacial defect states, like oxygen vacancies and pinholes, which are key sources of trap-assisted dark current under reverse bias [40]. This dual functionality significantly suppresses dark current, thereby enhancing device performance.

This strategy was shown to be universally applicable to SWIR PDs based on three distinct solution-processed photosensitive layers using organic and organic/inorganic hybrid materials systems. In a state-of-the-art CQD device with a spectral response ranging from 350 to 1650 nm, we achieved a two-order magnitude reduction in dark current, resulting in a D* of up to 4.3 × 10^12^ Jones at 1550 nm. Through integration with a CTIA-architecture ROIC, we successfully fabricated a megapixel SWIR imager with performance comparable to advanced InGaAs-based FPA detectors [41]. Fabricated through solution processing, this imager eliminates the need for complex epitaxy, etching and photolithography procedures, significantly reducing production costs and enabling scalability. This streamlined, versatile approach positions solution-processed PDs as a compelling alternative for high-performance and low-cost SWIR imaging.

## RESULTS

### Modification of the electrode by HIES

**Figure 1.**
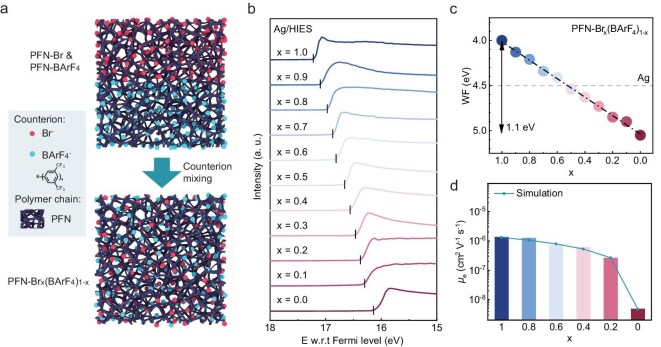
Characteristics of HIES materials. (a) Schematic diagram of the formation mechanism of HIES materials, using PFN-Br_x_(BArF_4_)_1−x_ as an example. Due to the mobility of counterions in the solution, the HIES can be obtained after counterion mixing. (b) UPS cutoff edge of Ag electrode modified with PFN-Br_x_(BArF_4_)_1−x_, illustrating shifts in the cutoff edge with varying x (Br^−^ proportion). (c) WF of Ag electrode modified with PFN-Br_x_(BArF_4_)_1−x_, derived from UPS measurements. The dashed gray line represents the WF of unmodified Ag. (d) Intrinsic electron mobility of PFN- Br_x_(BArF_4_)_1−x_ films.

The PFN and PNDIT-F3N material series are well-established CPEs, commonly used as electron transport layers (ETLs) in solar cells (SCs) and light-emitting diodes (LEDs) [42,43]. In these two material series, CPEs containing Br^−^ counterions and BArF_4_^−^ counterions exhibit opposite modification effects: Br^−^ decreases the WF of the metal substrate, while BArF_4_^−^ increases it [44]. CPEs with Br^−^ counterions have been confirmed to create electric dipole moment pointing opposite to the metal, which decreases the metal surface WF [45,46]. The steric hindrance of BArF_4_^−^ is proposed to induce localized dipoles, thereby causing its unique WF-increasing effect [39]. Integrating both counterions into CPEs while precisely controlling their ratio holds promise for achieving wide-range, precise tuning of the metal electrode surface WF. In polar solvents, the localized interactions between side chain groups and counterions in CPEs are disrupted, making counterions behave as quasi-free ions, which facilitates the control of mixing the different counterions [38,47]. Based on this, we developed a universal and straightforward counterion-mixing method (see Supplementary data for details) to synthesize two series of HIES materials, namely PFN-Br_x_(BArF_4_)_1−x_ and PNDIT-F3N-Br_x_(BArF_4_)_1−x_, as illustrated in Fig. 1a. The precursor CPEs with BArF_4_^−^ counterions were synthesized via a counterion exchange process from the precursor CPEs with Br^−^ counterion [39], ensuring the conjugated backbones and polydispersity of the CPEs remained unchanged. The resulting HIES materials also preserved these characteristics.

To evaluate the WF modulation ability of these HIESs, thin HIES films (∼5 nm) were deposited onto Ag electrodes and characterized using ultraviolet photoelectron spectroscopy (UPS). For PFN-Br_x_(BArF_4_)_1−x_, as the proportion of Br^−^ counterions (x) increased, UPS measurements showed a systematic reduction in WF, evidenced by a shift in the secondary electron cutoff toward higher binding energies (Fig. 1b and Fig. S1). The WF of these HIES-modified electrodes exhibited broad, linear tunability, ranging from 4.0 to 5.1 eV, with a strong correlation with the Br^−^ content (Fig. 1c). Consistent results were obtained via Kelvin probe force microscopy (KPFM), which revealed a similarly broad WF modulation range of 1.3 eV (Fig. S2a and S2b; Table S1). For PNDIT-F3N-Br_x_(BArF_4_)_1−x_, a WF modulation range from 3.8 to 5.0 eV was observed (Fig. S2c), and this analogous trend underscores the universality of this approach. From KPFM, smooth surface potential figures of modified electrodes were shown, further validating that this surface modification strategy enables uniform large-area modulation of the electrodes’ WF, with negligible patch-to-patch variations, even on scales smaller than 100 nm (Fig. S3). Additionally, the intrinsic properties of the HIES layers, including electron mobility and energy levels, were systematically tuned by varying the counterion ratio, as illustrated in Fig. 1d, Figs S4 and S5. The electron mobility of the HIES layer is directly proportional to the Br⁻ ion ratio x. Except when x is close to 0, the electron mobility of the HIES layer remains in the order of 10^−7^ to 10^−6^ over a wide range of x, with minimal variation. Notably, due to the ultra-thin nature of HIES layers, variations in electron mobility within these layers have a negligible impact on the electrode performance during the surface modification. These findings highlight the robustness of this straightforward surface modification strategy, enabling precise WF tuning without significantly altering other electrode characteristics. This capability offers a versatile and scalable solution for optimizing the performance of a wide range of semiconductor devices, including PDs, LEDs and SCs. By integrating tailored WF engineering, this approach provides a powerful platform for advancing electronic and optoelectronic applications.

### Configuration of PDs with HIES-modified anode

**Figure 2.**
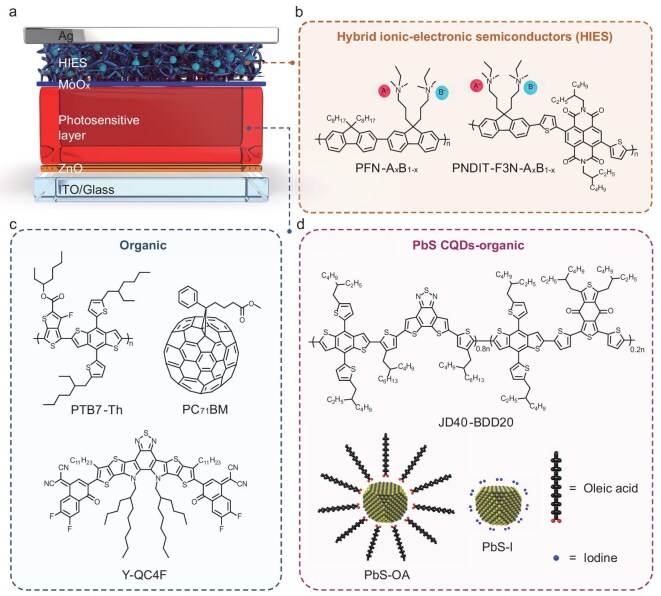
Solution-processed SWIR PDs with HIES. (a) Schematic illustration of the PD architecture. (b) Schematic diagram of HIES materials. A^−^ and B^−^ denote the two types of counterions, Br^−^ and BArF_4_^−^, respectively. The subscript x represents the proportion of A^−^ in the total counterion composition. (c and d) The materials used as photosensitive layer. PbS-OA CQD is the precursor of PbS-I CQD used as an effective photosensitive layer.

In diode-type PDs, the impact of interfacial states at the electrode remains insufficiently understood, often limiting device optimization. To address this, we introduced HIES layers as a research platform into a series of PDs with an n-i-p architecture (Fig. 2a and b), systematically evaluating their performance in three types of high-efficiency solution-processed systems, encompassing both organic and organic/inorganic hybrid photosensitive layers (Fig. 2c). The absorption spectra of these systems extend continuously from the visible to the NIR/SWIR regions, as shown in Fig. S6. These devices utilized a 40 nm zinc oxide (ZnO) layer as the ETL and a 10 nm molybdenum oxide (MoO_x_) layer as the HTL, both selected for their high charge transport efficiency and stability [40,48]. Control devices, without an HIES layer, exhibited performance metrics comparable to or exceeding previously reported values using the same photosensitive materials, serving as a reliable baseline for comparison [49–51]. The HIES layer was strategically introduced at the interface between the MoO_x_ and Ag anode. Since MoO_x_ is inherently a heavily n-type-doped semiconductor, which acts as an HTL with a recombination-type hole-extraction mechanism [40,52,53], inserting a thin or high-mobility n-type semiconductor layer between MoO_x_ and Ag via evaporation or solution processing does not significantly hinder hole extraction, as shown in Figs S7 and S8.

**Figure 3.**
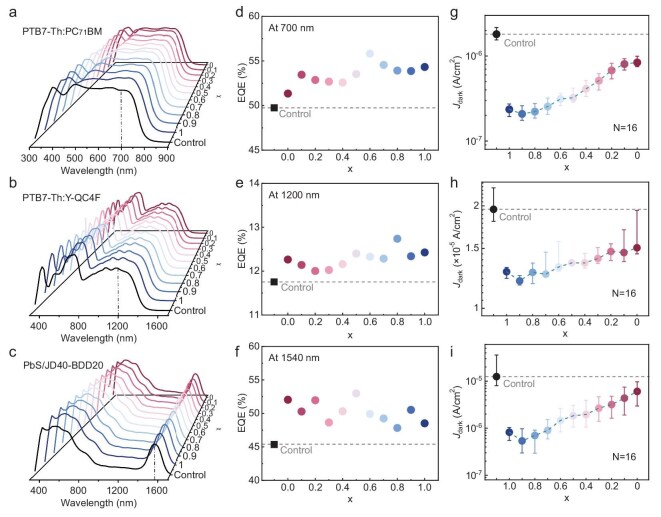
Performance of SWIR PDs with HIES. (a–c) The EQE–wavelength curves of PDs with an HIES. The dashed lines indicate the characteristic wavelengths of the PDs, which are used for comparison of EQE in (d–f). (d–i) EQE and *J*_dark_ of the PDs with an HIES compared to the control PDs, measured under −0.1 V bias. The sample size for each experimental group is 16. The photosensitive layers in these PDs are PTB7-Th:PC_71_BM (a, d and g), PTB7-Th:Y-QC4F (b, e and h) and PbS CQDs/JD40-BDD20 (c, f and i).

According to the Lambert–Beer law, the extremely thin and uniform HIES layers have minimal impacts on the light field distribution within the PDs. The negligible change in the shape of the response spectra, as shown in Fig. 3a–c, validates this point. After HIES treatment, the overall external quantum efficiency (EQE) of PDs based on PTB7-Th:PC_71_BM, PTB7-Th:Y-QC4F and PbS CQDs/JD40-BDD20 photosensitive layers was significantly enhanced (Fig. S9). Figure 3d–f shows the EQE of these PDs at their respective key wavelengths, with no obvious correlation between the counterion ratio of the HIES layer and EQE enhancement. In contrast, as shown in Fig. 3g–i, integration of the HIES layer in PDs effectively suppressed the dark-current density (*J*_dark_), with a clear correlation observed between these suppressions and the counterion ratio of the HIES layer. To avoid repetition, PDs based on PbS CQDs/JD40-BDD20 photosensitive layer were taken as the main object of discussion. A statistical analysis of *J*_dark_ at −0.1 V bias, conducted across multiple devices and independent repetitions (Fig. S10), consistently demonstrated lower *J*_dark_ in HIES-modified devices compared to control devices. As the Br^−^ counterion proportion (x) in PFN-Br_x_(BArF_4_)_1−x_ increases, *J*_dark_ gradually decreases, from 6.0 × 10^−6^ to 8.2 × 10^−7^ A cm^−2^. Notably, similar trends were observed in experiments involving different HIES series or PDs based on different photosensitive materials (Fig. 3g and h; Figs S11 and S12), which are likely attributed to the modulation of electron injection driven by the WF of the electrodes.

**Figure 4.**
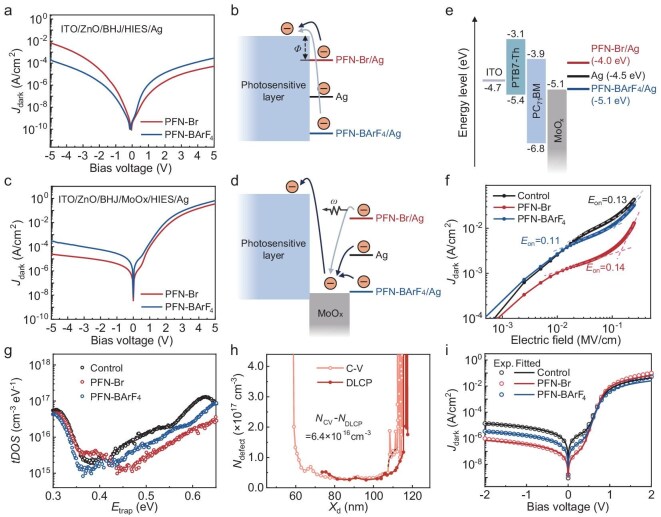
Mechanism of the HIES layer in PDs. (a) *J*_dark_–*V* curves of PTB7-Th:PC_71_BM devices with different interlayers, excluding the MoO_x_ layer. (b) The energy level diagram illustrating the electron injection from the Ag anode with surface modification by PFN-Br or PFN-BArF_4_. (c) *J*_dark_–*V* curves of PTB7-Th:PC_71_BM devices with different interlayers. (d) The corresponding energy level diagram demonstrating how the PFN-Br-modified Ag anode WF impacts electron transport efficiency due to momentum mismatch with MoO_x_. (e) Energy level diagram and (f) *J*_dark_–electric field characteristics of devices in electron injection measurement, highlighting the *E*_on_ variations with and without an HIES layer. (g) Comparison of *tDOS* in devices with and without HIES interlayers, obtained via admittance spectroscopy measurements. (h) Defect density profiles of devices with a PFN-Br interlayer, showing depletion width (*X*_d_) and defect densities (*N*_CV_, *N*_DLCP_) obtained from *C–V* and DLCP measurements, respectively. (i) Experimental (solid lines) and fitted (dashed lines) *J*_dark_–*V* curves for devices with and without HIES interlayers.

To deepen understanding of the electron injection mechanism, we fabricated devices with an indium tin oxide (ITO)/ZnO/photosensitive layer/HIES/Ag structure, excluding the MoO_x_ layer. The *J*_dark_–*V* characteristics of these devices, based on PTB7-Th:PC_71_BM and with PFN-Br and PFN-BArF_4_ as the HIES layer, were compared with those of control devices, as shown in Fig. 4a. In the absence of MoO_x_, the *J*_dark_ under reverse bias predominantly reflected the directed electron injection from the Ag electrode to the photosensitive layer. As depicted in Fig. 4b, devices with PFN-Br exhibited a lower electron injection barrier (*Φ*) compared to devices with PFN-BArF_4_. This lower barrier facilitated electron injection, subsequently leading to an increase in the *J*_dark_ under reverse bias. However, introducing an MoO_x_ layer reversed this trend: under reverse bias, PFN-Br devices exhibited lower *J*_dark_ than PFN-BArF_4_ devices (Fig. 4c), which cannot be explained solely by *Φ*. Through detailed systematic analysis (see Notes in Supplementary Data), we further confirm that classical surface-related potential factors, such as localized states, image charge effects and interface dipoles, are not the primary causes of the experimental phenomena observed in this work. Instead, it suggests a more complex interlayer electron transfer process from Ag to MoO_x_, likely influenced by momentum mismatch [54–56]. Although electrons move from a high energy level to a low energy level, there exists a significant mismatch in both energy and momentum between the conduction electrons in PFN-Br/Ag and the electronic states in the MoO_x_ conduction band (Fig. S13). To satisfy momentum conservation, this mismatch necessitates quasi-phonon (*ω*) assistance during the transition process. It is important to note that in amorphous materials, there is no consistent ‘phonon’ derived from the collective lattice vibration modes as defined in semiconductor physics. However, similar to phonons, there are coupling effects between structural vibrations and electrons in amorphous materials, which have been reported [57–59], and can be referred to as ‘quasi-phonon’. The involvement of quasi-phonons reduces the probability of electron transfer and slows down the process [60], thereby hindering efficient electron transition from Ag into MoO_x_ (Fig. 4d). As a result, this mechanism increases the injection difficulty and effectively suppresses the *J*_dark_ under reverse bias and dark conditions.

Further investigation into electron injection dynamics was conducted in devices with an ITO/PTB7-Th:PC_71_BM/MoO_x_/HIES/Ag configuration (Fig. 4e). As illustrated in Fig. 4f, the turn-on electric field (*E*_on_) under reverse bias can be determined from the *J*_dark_–*V* curves, and also correlates with electron injection efficiency [61]. Devices with PFN-Br exhibited a higher *E*_on_ of 0.14 MV cm^−1^ compared to both the control devices (0.13 MV cm^−1^) and PFN-BArF_4_ devices (0.11 MV cm^−1^). This higher *E*_on_ indicates that it is more challenging for electrons to be injected from the Ag electrode into the devices under reverse bias. This observation underscores the impact of increasing the mismatch between the WF of the Ag electrode and the electron affinity of MoO_x_, which decreases electron injection efficiency and highlights the critical role of WF modulation in controlling electron injection behavior.

Notably, two intriguing phenomena emerged from the statistical analysis of *J*_dark_. First, the lowest *J*_dark_ did not occur at x = 1 (corresponding to the lowest electrode WF). Second, in the range of x = 0.5–0.6, where the WF of the modified Ag electrode equals that of the unmodified Ag electrode, the HIES devices exhibited an obviously lower *J*_dark_ of 1.4 × 10^−6^ A cm^−2^, which is approximately 10-fold lower than that of the control device (1.3 × 10^−5^ A cm^−2^). These anomalies indicated that the performance improvements induced by the HIES layer are not solely due to modulation in electrode WF but involve additional interfacial effects.

### Interfacial defect passivation in PDs with HIESs

The introduction of HIESs at the MoO_x_/Ag interface, while leaving the rest of the PD structure unchanged, suggests that the observed performance improvements primarily stem from the modification of the interfacial state. To verify this hypothesis, defect analysis was conducted using capacitance–frequency (*C–f*) and capacitance–voltage (*C–V*) measurements (Fig. S14), which revealed a significant reduction in trap density of states (*tDOS*) for HIES-modified devices (Fig. 4g). In the shallow trap energy region ranging from 0.30–0.35 eV, the *tDOS* decreased by 0.6 × 10^16^ cm^−3^ eV^−1^ for PFN-Br devices and 1.3 × 10^16^ cm^−3^ eV^−1^ for PFN-BArF_4_ devices when compared to controls. This decrease in *tDOS* leads to a reduction in thermal activation under dark conditions [62]. For deep traps within the energy range of 0.45–0.65 eV, significant reductions in the *tDOS* were observed. Specifically, decreases of 3.5 × 10^16^ cm^−3^ eV^−1^ for PFN-Br devices and 2.2 × 10^16^ cm^−3^ eV^−1^ for PFN-BArF_4_ devices were observed. These reductions are associated with the suppression of trap-assisted recombination and reducing thermal noise [25,63], as illustrated in Fig. S15. Gaussian fitting of peaks in the 0.35–0.45 eV range showed that PFN-BArF_4_ achieved superior trap suppression compared to PFN-Br (Table S2).

Steady-state *C–V* measurements further revealed that the devices with an HIES layer exhibited enhanced built-in voltage and expanded depletion regions compared to the control devices (Table S3). This promotes more efficient photogenerated exciton dissociation in devices with an HIES, which explains the observed enhancement in EQE. *C–V* and drive-level capacitance profiling (DLCP) measurements can provide the spatial distribution of defects within the devices. Significantly, due to the varying sensitivities of *C–V* and DLCP measurements to interface defects, comparing the results of these two measurements can provide a more comprehensive analysis of the interface defects. Defect density profiling via *C–V* and DLCP (Fig. 4h, Figs S16 and S17) confirmed a lower interfacial defect density of 6.4 × 10^16^ cm^−3^ for PFN-Br devices and 6.3 × 10^16^ cm^−3^ for PFN-BArF_4_ devices exhibited in HIES-modified devices than that of 8.3 × 10^16^ cm^−3^ for control devices, which is consistent with the results of *tDOS* [13,64]. The consistent trends validate the better interfacial defect passivation capabilities of PFN-BArF_4_ compared to PFN-Br.

Electrical analysis of *J*_dark_*–V* characteristics fitted using the non-ideal generalized Shockley diode equation is shown in Fig. 4i, Fig. S18 and Table S4. The ideality factor (*n*) primarily reflects the quality of the main p–n junction and the non-radiative recombination loss. Given that HIES modification does not substantially alter the characteristics of the main p–n junction, the slight variations in *n* can be reasonably attributed to interfacial optimization, which reduces overall non-radiative recombination losses in the diode. The reduction in saturation current density (*J*_0_) further indicates that HIES modification can enhance the overall diode quality. The optimized devices exhibit lower ohmic leakage current density and higher shunt resistance (*R*_sh_) than that of control devices, which is consistent with reduced thermal noise ($\\lesssimngle {i_{{thermal}}^2} \rangle \ \approx 4kT/{R}_{sh}$) [65]. The observed suppression of ohmic leakage is attributed to the incorporation of the thin HIES layer with low carrier mobility, which reduces the probability of direct tunneling and fills pinhole-like low-resistance channels at the MoO_x_/Ag interface [66,67]. Devices incorporating HIES exhibit lower trap-assisted tunneling current density (*J*_TAT_) compared to control devices, consistent with a reduced interface trap state density. The parameter *B*, which reflects the energetic depth of trap states within the bandgap, remains essentially unchanged, whereas parameter *A* decreases, aligning well with the capacitance measurement results. The reduction in *A* further confirms that PFN-BArF_4_ more effectively suppresses trap states than PFN-Br.

Given their identical polymer backbones, the difference in interfacial defect passivation between PFN-Br and PFN-BArF_4_ is likely attributable to their different counterions. Comparative analysis suggests that the larger ionic volume of BArF_4_^−^ could contribute to its superior passivation effect. To test this hypothesis, Blm_4_^−^ was selected as a contrasting counterion to Br^−^. According to previous reports, Blm_4_^−^ and Br^−^ exhibit comparable capabilities in reducing electrode WF [44], thereby allowing us to isolate the effect of WF modulation on device performance. As shown in Fig. S19, Blm_4_^−^ possesses a larger volume than Br^−^ and a molecular structure analogous to that of BArF_4_^−^. We compared the influence of Br^−^ and Blm_4_^−^ on device performance in both PFN and PNDIT-F3N systems. In both cases, devices incorporating Blm_4_^−^ exhibited lower *J*_dark_ and higher EQE values than their Br⁻ counterparts (Fig. S19), consistent with our hypothesis. These results indicate that larger counterion volume exhibits greater potential for interfacial defect passivation, with similar conclusions reported in other fields [68].

These findings confirmed that the HIES layer can effectively passivate interfacial defects at the MoO_x_/Ag interface, thereby reducing recombination and finally contributing to the observed enhancement in EQE. The observed reductions in *J*_dark_ are attributed to the concurrent effects of electrode WF modulation and interfacial defect passivation (detailed mechanism analysis shown in Fig. S20). HIES materials incorporating BArF_4_^−^ demonstrated superior defect passivation capabilities compared to those with Br^−^, providing a plausible explanation for the observed anomalies in the *J*_dark_ trends from previous gradient experiments. Furthermore, beyond CPE-type HIESs, small-molecule HIESs have also been shown to enhance detector performance, further supporting the general applicability of this interfacial engineering strategy (Fig. S21). However, it should be noted that, based on a comparative analysis of HIES modification across different systems and supported by previous findings (Figs S18, S22 and S23; Tables S4–S6), this strategy has limitations. While it effectively suppresses the injection under reverse bias and reduces the interfacial trap states at the electrode/functional layer interface, it does not substantially improve the intrinsic quality of the diode’s main junction. As a result, its effectiveness is limited in devices where the dark current is primarily governed by the main junction rather than by interfacial effects, especially when the trap-state density in the main junction is excessively high.

### Performance of HIES-optimized SWIR PDs

**Figure 5.**
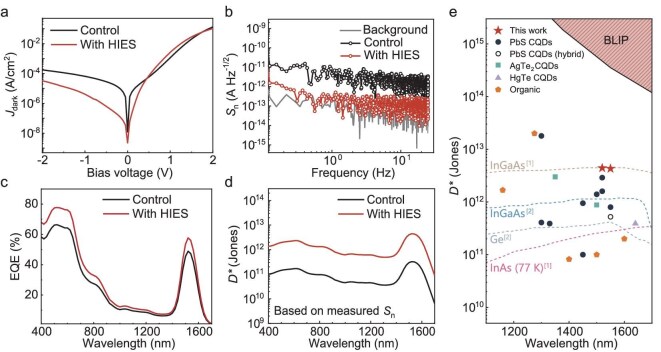
Impact of interfacial modification on SWIR PD performance. (a) *J*_dark_–*V* curves, (b) *S*_n_–*f* curves, (c) EQE and (d) D* for SWIR PDs based on PbS CQDs/JD40-BDD20 photosensitive layer. *S*_n_ and EQE are measured under −0.1 V bias. D* values are obtained from *S*_n_ at 10 Hz (B = 1 Hz) under −0.1 V bias. (e) Comparison of the D* values of optimized SWIR PDs with reported solution-processed SWIR PDs and the commercial inorganic SWIR PDs. The black line shows the background-limited infrared performance (BLIP) of PDs operating at a temperature of 300 K. Colored dashed lines indicate commercial PDs with data sourced from ^[1]^Hamamatsu [69] and ^[2]^Thorlabs [18]. Data without special instructions indicate that the device testing environment was sustained at room temperature.

By incorporating an HIES layer for interfacial modification, we achieved solution-processed SWIR PDs based on an organic/inorganic hybrid photosensitive layer with exceptional performance. Under reverse bias, the noise current (*i*_noise_) is dominated by shot noise (*i*_shot_), which is proportional to the square root of the dark current (${i}_{\mathrm{shot}} = \sqrt {2q{i}_{\mathrm{dark}}}$). As depicted in Fig. 5a, the HIES modification significantly suppressed the *J*_dark_ of SWIR PDs under reverse bias. Notably, the optimized devices demonstrated a dramatically lower *J*_dark_ of 6.7 × 10^−8^ A cm^−2^ at −0.1 V, which is almost two orders of magnitude lower than that of control devices (5.4 × 10^−6^ A cm^−2^). As shown in Fig. S23 and Table S6, fitting analysis using the non-ideal generalized Shockley diode equation reveals that the primary mechanism for dark-current suppression in the optimized devices is the reduction of ohmic leakage. Concurrently, the *J*_TAT_ component is reduced to less than half its original value, and *J*_0_ decreases from 1.7 × 10^−7^ to 1.2 × 10^−8^ A cm^−2^—consistent with effective defect passivation and a lowered interface trap state density. This reduction in *J*_dark_ led to a one-order-of-magnitude lower spectral density of *i*_noise_ (*S*_n_) across all frequencies within the range of 0.1 Hz to 102.4 kHz (Fig. 5b and Fig. S24). As shown in Fig. S25, the HIES-mediated suppression of *J*_dark_ is identical in devices with sputtered ITO or Ag top electrodes, confirming that HIES withstands high-energy particle bombardment during sputtering. This highlights the broad adaptability of HIES to typical semiconductor fabrication techniques. Additionally, the spectral responses of SWIR PDs have exhibited a significant improvement after HIES modification, achieving an EQE of 57.7% and a responsivity (*R*) of 0.71 A W^−1^ at the 1550 nm characteristic wavelength, as illustrated in Fig. 5c and Fig. S26. These improved response capabilities originate from the enhanced photogenerated exciton dissociation and suppressed trap-assisted recombination. Due to the lower *S*_n_ and optimized carrier transport, HIES devices maintain a linear response at lower light intensities, and exhibit saturation at higher light intensities compared to control devices, thereby exhibiting a broad linear dynamic response range (*LDR*), which is sufficiently comparable to that of commercial InGaAs detectors (Fig. S27). The *LDR* value calculated using the formula reported in the materials field [9,13] exceeds 130 dB. However, it is worth noting that, due to flaws in the derivation of this formula, this value represents a significant overestimation compared with the value (65 dB) calculated based on the definition in engineering. Therefore, attention should be paid to its calculation method when conducting horizontal performance comparisons. Fast response times (*t*_rise_/*t*_fall_ = 1.54 μs/2.89 μs) and a high cutoff frequency of 107 kHz were also achieved, as shown in Fig. S28. Based on extrapolating the signal-to-noise ratio (SNR) to unity (Fig. S29), the noise equivalent power (NEP) under a 1550 nm light source was calculated as 254 fW Hz^−^^½^, which is close to the calculated value of 219 fW Hz^−^^½^ using the formula $NEP = {i}_{\rm n,rms}/( {R\sqrt B } )$, where *i*_n, rms_ is the root mean square noise obtained from *S*_n_, and *B* is the measured bandwidth (1 Hz). For a comprehensive assessment of the detection performance [65], the dependences of D* (based on measured *S*_n_) on both frequency and wavelength were analyzed (Fig 5d and Fig. S30). The D* of HIES-modified devices achieved a D* of 4.4 × 10^12^ and 4.3 × 10^12^ Jones at 1520 nm (the peak wavelength) and 1550 nm, respectively, outperforming current solution-processed SWIR PDs and approaching the performance of advanced commercial InGaAs SWIR PDs (Fig. 5e and Table S7). These advancements demonstrate that solution-processed SWIR PDs, enhanced with an HIES layer, not only exhibit superior detection performance but also hold promise for integration with high-resolution readout arrays, making them a viable alternative for cost-effective, high-performance imaging applications.

### Implementation of SWIR FPA imagers and applications

**Figure 6.**
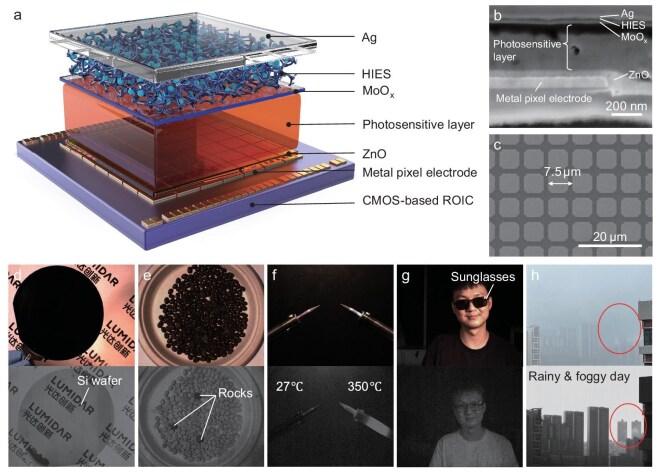
SWIR FPA imagers based on solution-processed PDs. (a) Schematic illustration of the solution-processed PDs integrated with a CMOS ROIC. (b) Cross-sectional SEM image of an individual pixel showing the vertical structure. (c) SEM image of the electrode array fabricated on the CMOS substrate. (d–h) Comparative analysis of images captured using a commercial visible light camera (MV-CU004-10GC, Hikrobot) and the developed solution-processed-based SWIR camera. Top row: visible camera images; bottom row: SWIR camera images. (d) Visualization of internal structures through a silicon wafer. (e) Discrimination of rocks concealed among coffee beans. (f) Detection of temperature gradients in hot iron. (g) Recognition of facial features under sunglasses occlusion. (h) Imaging in adverse weather conditions, including fog and rain.

To validate the SWIR imaging capabilities, we developed SWIR FPA imagers integrated with CMOS ROICs. The CMOS ROICs employ a high-sensitivity architecture with small capacitance and high gain, requiring PDs with low dark-current to achieve a high SNR. However, the advancement of solution-processed SWIR FPA imagers has been hindered by the high dark-current and noise of existing SWIR PDs at room temperature. To address this, we vertically integrated our optimized, low-noise SWIR PDs onto CMOS ROICs featuring a 1280 × 1024-pixel array in a top-illuminated configuration (Fig. 6a) and finally achieved the first solution-processable SWIR imager based on organic semiconductors. The fabrication process is fully compatible with CMOS manufacturing, eliminating the need for costly and complex patterning, etching and flip-chip bonding (Fig. S31). Detailed fabrication methods and architectural schematics are provided in the Supplementary data. A cross-sectional scanning electron microscopy (SEM) image (Fig. 6b and Fig. S32) illustrates the precise vertical structure of an individual pixel and the continuous photosensitive layer that obviates the need for pixel-level patterning. The underlying ROIC employs a sophisticated three-transistor (3T) architecture with a 7.5 μm pixel pitch (Fig. 6c). Incident light is absorbed by the photosensitive layer, generating excitons that dissociate into electrons and holes at the interface. The resulting electrons are collected and processed by the ROIC for subsequent signal readout. Electrical integration of CMOS electrodes with external circuitry was achieved via gold wire bonding, enabling real-time image acquisition at 60 fps.

The SWIR imager was rigorously evaluated in comparison with a commercial visible light camera (MV-CU004-10GC, Hikrobot) under diverse scenarios (Fig. 6d–h). SWIR images, displayed without any post-processing, were taken in a controlled environment illuminated by a halogen lamp and filtered for SWIR wavelengths. For instance, Fig. 6d shows silicon wafers that appear opaque under visible light but exhibit transparency in SWIR, enabling detailed visualization of internal structures critical for semiconductor inspection (Fig. S33). Similarly, Fig. 6e highlights the ability of SWIR imaging to distinguish stones hidden among coffee beans, a feat impossible with a visible light camera. Other applications include non-invasive food inspection through opaque packaging (Fig. S34) and the precise detection of temperature gradients and material textures during low-temperature welding processes (250°C–500°C, Fig. 6f). SWIR imaging also proved to be effective for facial recognition and security, with clear visualization of facial features through sunglasses, even under natural light (Fig. 6g and Fig. S35). Furthermore, its robustness in adverse weather conditions, such as fog and rain, enabled imaging clarity at a distance of over 1 km, demonstrating significant potential for autonomous navigation and maritime defense applications (Fig. 6h). Notably, we benchmarked the imaging performance of our SWIR imager against a commercial InGaAs-based device (GH-SW640-U2, GHOPTO). As shown in Fig. S36, under illumination with an SWIR light source of identical intensity, our device—with its extended dynamic response range—more clearly resolved high-reflectance regions, whereas the InGaAs imager suffered from widespread circular saturation artifacts. Our imager also captured significantly richer detail across the visible-to-SWIR spectrum, owing to its broader spectral responsivity. The comparison of the modulation transfer function (MTF) analysis based on ISO 12233 resolution test chart (Figs S37 and S38), conducted under identical conditions (integration time *t* = 5 ms; gain = 1), revealed that our imager delivered higher MTF50 and sharper images than those of commercial InGaAs cameras despite having the same pixel resolution. This enhanced spatial clarity is likely attributed to suppressed optical and electrical crosstalk (Fig. S39), enabled by the relatively thinner photodetector layer. Collectively, these results underscore the transformative potential of solution-processed SWIR imagers for an extensive range of industrial, medical and defense-related applications.

## DISCUSSION

This study demonstrates that HIES layers effectively suppress dark current in solution-processed SWIR PDs by tuning the metal anode WF (4.0–5.1 eV) through optimized Br^−^:BArF_4_^−^ counterion ratios and passivating the defects at the anode/semiconductor interface. The HIES interlayer delivers substantial performance enhancements across a range of solution-processed semiconductor materials, including organic and organic/inorganic hybrid systems. In organic/inorganic hybrid systems, HIES interlayers enable a reduction in dark current by up to two orders of magnitude under −0.1 V bias, while maintaining high charge extraction efficiency, leading to a superior D* of up to 4.3 × 10^12^ Jones at 1550 nm, a high *LDR* and a −3 dB bandwidth above 107 kHz. Notably, the seamless integration of HIES-modified SWIR PDs with CMOS ROICs, resulting in high-resolution 1280 × 1024-pixel imagers, underscores the practical viability of this technique for next-generation SWIR imagers. Beyond addressing fundamental limitations such as dark current and scalability, this work highlights the broader impact of HIES-modified SWIR PDs as cost-effective, high-performance alternatives to traditional semiconductor counterparts, offering a scalable and versatile platform to meet the growing demand for state-of-the-art SWIR imaging solutions across a wide range of imaging applications.

## METHODS

Detailed materials and methods are available in the online Supplementary data.

## Supplementary Material

nwaf531_Supplemental_Files
