# Supplementary material for: Hybrid ionic–electronic semiconductors for interface engineering of ultra-low-dark-current solution-processed SWIR photodetectors
Source: Natl Sci Rev. 2025 Nov 26;13(2):nwaf531. doi: 10.1093/nsr/nwaf531 (PMC12831028; doi:10.1093/nsr/nwaf531)
Supplement: nwaf531_Supplemental_Files [file nwaf531_supplemental_files.zip › Supplementary data.pdf]

Supplementary Data

Hybrid ionic-electronic semiconductors for interface engineering of ultra-low-dark-current solution-processed SWIR photodetectors

Yunhao Cao[1], Xiye Yang[2], Yazhong Wang[1,2], Jingwen Chen[1], Haoran Tang[1], Chunchen Liu[1], Kai Zhang[1], Sheng Dong[2], Yong Cao[1], Fei Huang[1,*]

[1]State Key Laboratory of Luminescent Materials and Devices, Guangdong Basic Research Center of Excellence for Energy & Information Polymer Materials, Institute of Polymer Optoelectronic Materials and Devices, School of Materials Science and Engineering, South China University of Technology, Guangzhou 510640, P. R. China;

[2]Lumidar Technology Co., Ltd.; Guangzhou, 510530 P. R. China

*Corresponding author. E-mail: msfhuang@scut.edu.cn.

1. Experimental Section

Materials preparation

Unless otherwise stated, all solvents and chemical reagents were commercially purchased and used as received without further purification. The compounds Y-QC4F, JD40-BDD20, P1, PFN-Br, PFN-BArF$_4$, PNDIT-F3N-Br and PNDIT-F3N-BArF$_4$ were synthesized following previously reported methods [39,50,70–72]. For HIES, we pre-prepared completely dissolved PFN-Br and PFN-BarF$_4$ or PNDIT-F3N-Br and PNDIT-F3N-BarF$_4$ (c = 0.5 mg mL^{-1}), and then mixed them at room temperature for more than 1 h according to the volume ratio calculated based on the proportion of corresponding counterion groups. When CPEs are dissolved in polar solvents, counterions delocalize to promote counterion mixing. After film formation, the localized interactions between counterions and side-chain groups are reconstructed, such that each polymer repeating unit hosts one counterion. The dipole formation induced by counterions remains independent of counterions from adjacent units, enabling macroscopic observation that the electrode work function is tuned by the counterion ratio. PbS CQDs with oleic acid (OA) long-chain ligands were synthesized from lead oxide (PbO) and bis(trimethylsilyl) sulfide by the typical Hines synthesis method [73]. The resulting PbS-OA and the short-chain ligand tetrabutylammonium bromide (TBAI) were dissolved in octane and methanol, respectively. Precursor solutions were stirred for at least 2 hours in a nitrogen-filled glove box and filtered through a 0.22 μm PTFE membrane filter before using.

Active layer deposition

The active layer (~ 200 nm) of PTB7-Th:PC$_{71}$BM (c = 9:13.5 mg mL^{-1} in chlorobenzene (CB) with 3%vol octamethylene diiodide) and PTB7-Th:Y-QC4F (c = 10:15 mg mL^{-1} in CB with 2%vol 1-chloronaphthalene) were obtained by spin-coating.

The active layer (~ 120 nm) of PbS CQDs was fabricated by repeating the following sequences in a loop 4 times: a) depositing PbS-OA CQDs (c = 50 mg mL^{-1}) at 2000 rpm for 15 seconds (ramping time of 1 second); b) covering the PbS-OA CQDs layer with TBAI solution (c = 10 mg mL^{-1}) for 30 seconds ligand exchange; c) cleaning the film with MeOH twice to remove the long-chain ligand. Then a 30 nm layer of JD40-BDD20 (c = 4 mg mL^{-1} in CB) was spin coated dynamically after the PbS CQDs deposition.

Device fabrication

For PDs, the ITO-coated glass substrates were cleaned sequentially via ultrasonication in isopropanol, deionized water with 1%vol detergent solution, deionized water, and isopropanol. The substrates were dried at 60°C for 5 hours. The sol-gel ZnO was prepared by dissolving 0.4 g zinc acetate in 4 mL 2-methoxyethanol with 110 μL ethanolamine, stirring at 50ºC for 12 h. The ZnO film (~ 40 nm) was spin-coated onto the substrate and thermally annealed at 150°C for 30 minutes. The active layer was then deposited onto the ZnO film, followed by thermal annealing at 80°C for 10 minutes in a nitrogen-filled glove box. A 10 nm MoO_x film layer was deposited by thermal evaporation under a vacuum of 9×10^{-7} torr, followed by spin-coating a HIES layer (c = 0.5 mg mL^{-1} in methanol). Finally, a 100 nm silver (Ag) electrode was thermally evaporated under the same vacuum condition. Devices had an effective device area of 0.0516 cm^2, defined using a shadow mask with four devices per substrate. For SWIR focal plane array (FPA) imager chips, the PDs were fabricated onto clean complementary metal-oxide-semiconductor (CMOS) readout integrated circuit (ROIC) chips using a process similar to that previously detailed. The top Ag electrode was thinned to ensure the incidence of the illuminated light. The layers on the edge electrode of CMOS were removed by laser to expose the electrodes, facilitating the subsequent connection of CMOS with external circuits.

SWIR FPA imager fabrication

The CMOS ROIC chips were provided by Lumidar Technology Co., Ltd. The fabrication of the SWIR FPA imager chips followed previously described methods. The chips were packaged, and wire bonding was used to connect them to the contact pads inside the packages. The packages were then sealed with sapphire covers. Finally, the completed packages were integrated with an external control system to finalize the SWIR FPA imagers.

External quantum efficiency (EQE)

EQE was measured using a commercial system (Ruoshui Co., Ltd.) equipped with calibrated silicon (Si) and indium gallium arsenide (InGaAs) detectors.

Current density-voltage (*J-V*) curves

J-V characteristics were recorded using a Keithley 2450 source-meter under dark condition in a nitrogen atmosphere.

Ultraviolet photoelectron spectroscopy (UPS) and reflected electron energy loss spectroscopy (REELs)

The UPS and REELs measurements were conducted using a Thermo Fisher ESCALAB XI+. For the UPS measurement, the energy source was HeIα at 21.22 eV. A bias voltage of −5 V was applied for work function (WF) measurements, while no additional voltage was used for valence band edge analysis. REELS employed an electron beam to detect energy loss, enabling determination of the semiconductor's electronic bandgap.

Absorption spectra

Absorption spectra were acquired using a SHIMADZU UV-3600i Plus spectrophotometer. Samples were deposited on quartz substrates, and blank quartz substrates were used for baseline calibration.

Thickness measurement

Layer thicknesses (excluding HIES layers) were measured with a Dektak 150 profilometer. The HIES layer thicknesses were determined using absorption spectra. Initially, a thick film (> 50 nm) was measured with a profilometer for calibration. Its absorption spectrum served as a reference to calculate thin film thicknesses by comparing absorbance peak intensities, following the Lambert-Beer law.

Kelvin probe force microscopy (KPFM)

KPFM measurements were performed using a Bruker Dimension Icon atomic force microscope. Samples were deposited on grounded silicon substrates, with all biases applied to the tip during the measurements.

Space-charge limited current (SCLC)

Electron-only devices with the architecture ITO/ZnO/HIES layer/C_{60}/Ag were fabricated. *J*-*V* characteristics under dark condition were measured using a Keithley 2450 source-meter. Electron mobility was calculated by fitting the dark current to the single-carrier SCLC model described by the equation:

$$J = \frac{9}{8}\varepsilon_0\varepsilon_r\mu\frac{V}{d^3} \quad (1)$$

where J is the current denesity, μ is the mobility, ε_0 is the vacuum permittivity, ε_r is the relative permittivity of the material, V is the effective voltage, and d is the thickness of the organic layer. The effective voltage can be obtained by subtracting the built-in voltage (V_{bi}) and the voltage

drop (V_s) from the substrate's series resistance from the applied voltage (V_{appl}), $V = V_{appl} - V_{bi} - V_s$. The electron mobility can be evaluated from the slope of the $J^{1/2}$-V curves.

Capacitance spectra

Capacitance-frequency (C-f) spectra were performed from 10 MHz to 1 Hz under 0 V bias and dark condition. The capacitance-voltage (C-V) and C-f spectra were measured using the PAIOS platform equipped with a Keysight E4990A impedance analyzer. For both measurements, an AC voltage of 100 mV was applied. C-V spectra were recorded within a voltage range of −2 V to 2 V under dark condition, with a frequency of 10 kHz. C-f spectra were measured across a frequency range from 10 MHz to 1 Hz at 0 V bias in a dark environment.

Drive-level capacitance profiling (DLCP)

The DLCP measurement were conducted using the the PAIOS platform. The DC bias (V_{DC}) was scanning from −1 V to 0.7 V in 20 mV steps, while the amplitude of the AC biases (V_{AC}) were ranging from 20 to 200 mV also in 20 mV steps. For each V_{AC}, the maximum forward bias voltage (V_{DC}+V_{AC}) was kept constant.

Simulation of J_{dark}-V curves

The J_{dark}-V of ideal diode should follow the Shockley diode equation:

$$J_{dark} = J_0\left[e^{\frac{q(V-J_{dark}R_s)}{nkT}} - 1\right] \tag{2}$$

Where J_0 is the saturation current density, R_s is the series resistance, n is the quality factor of the p-n junction, q is the electron charge, k is the Boltzmann constant and T is the temperature. However, real diodes also exhibit ohmic leakage currents density (J_{ohmic}) caused by direct tunneling, pinholes and local geometric defects, as well as trap-assisted tunneling currents density (J_{TAT}) induced by strong electric fields. Therefore, they can be fitted using the non-ideal generalized Shockley diode equation:

$$J_{dark} = J_0\left[e^{\frac{q(V-J_{dark}R_s)}{nkT}} - 1\right] + \frac{V - J_{dark}R_s}{R_{sh}} + AVe^{\frac{B}{V-V_{bi}}} \tag{3}$$

Where R_{sh} is the shunt resistance, A and B are the factors related to the trap density and trap state energy level. The first two terms are used to perform a preliminary fitting on the data (V > −0.5 V), and then the results are taken as the initial values for fitting the complete equation.

Spectral density of the noise current (S_n)

The S_n were measured under dark condition. Current signals were amplified using a low-noise current preamplifier (SR 570) and digitized using a dynamic signal analyzer (Stanford Research, SR785) with a fast Fourier transform (FFT).

Linear dynamic range (*LDR*)

The *LDR* was measured under varying 1550 nm illumination intensities, calibrated using an optical power meter (Newport 1919-R) paired with an InGaAs detector (Newport 818IG/DB). Transient response measurements were captured with a digital oscilloscope (Tektronix TDS3052B), and input signals were amplified using a current preamplifier (SR 570). The 1550 nm light source comprised a light-emitting diode (LED) and a laser (Changchun New Industries Optoelectronics Tech. Co., FC-W-1550-15W). When *LDR* was calculated using the equation [9,13]:

$$LDR = 20\log\left(\frac{J_{ph,max}}{J_{ph,min}}\right) = 20\log\left(\frac{P_{\mathrm{max}}}{P_{\mathrm{min}}}\right) \tag{4}$$

where P_{max} (P_{min}) is the highest (lowest) incident optical power in the linear region where the slope of the J_{ph}-P curve is 1. Meanwhile, ensure that R remains essentially constant within this region. The derivation in the equation (4) is based on the premises that $J_{ph} = R \times P_{opt}$ and R is a constant. It is noteworthy that equation (4) is inconsistent with the definition of *DR* in engineering. According to the engineering definition, LDR should be given by the following equation:

$$LDR = 10\log\left(\frac{P_{max}}{P_{min}}\right) = 10\log\left(\frac{J_{ph,max}}{J_{ph,min}}\right) \tag{5}$$

Using the aforementioned different equations to calculate *LDR* will lead to numerical discrepancies. Consequently, when comparing device performance, care should be taken to consider the calculation method employed by the data source.

Noise equivalent power (NEP)

The NEP was measured by gradually decreasing the illumination intensity of a 1550 nm light source, modulated at 10 Hz. The intensity was calibrated using an optical power meter (Newport 1919-R) coupled with an InGaAs detector (Newport 818IG/DB). The signal-to-noise ratio (SNR) was recorded using a system consisting of a preamplifier and a dynamic signal analyzer (with a resolution bandwidth set to 1 Hz). The SNR was then plotted as a function of input light power. The NEP is determined at the point where the SNR equals to unity, corresponding to the optical power of the light source.

Cutoff frequency (f_{-3dB}) and response speed

The f_{-3dB} and response speed were measured using a low-noise current preamplifier (SR 570) and a mixed-domain oscilloscope (Tektronix MDO3052). A 1550 nm LED was modulated with a square wave signal generated by an arbitrary function generator (Tektronix AFG1062).

Focused ion beam scanning electron microscope (FIB-SEM)

The top and cross-sectional views of the SWIR FPA imager were obtained using FIB-SEM (TESCAN, GAIA3 and Thermoscientific, Helios 5 UX). The top view was captured at a work distance of 5.07 mm with a test voltage of 15 kV. For the cross-sectional view, the pixel cross-section was milled at an angle of 55° using FIB, followed by imaging at an angle of 65° with a test voltage of 3 kV.

Relative dielectric constants (ε_r) and depletion width (*W*)

The ε_r were determined by the *C*-*V* measurements performed on a device structure of ITO/test film/Ag and calculated from the following equation:

$$\varepsilon_{\mathrm{r}} = \frac{Cd}{\varepsilon_0} \tag{6}$$

where *C* is the capacitance, *d* is the thickness of the organic layer, ε_0 is the vacuum permittivity, and *A* is the effective device area (0.0516 cm^2 in this work).

The depletion width (*W*) can be described by following equation:

$$W = \sqrt{\frac{2\varepsilon_0\varepsilon_{\mathrm{r}}(V_{\mathrm{bi}} - V)}{qN}} \tag{7}$$

where *N* is the defects density.

Density of trap state (*tDOS*)

The *tDOS* can be calculated from *C*-*f* spectra by using following equation:

$$DOS(E_\omega) = -\frac{1}{qkT}\frac{\omega dC}{d\omega}\frac{V_{\mathrm{bi}}}{W} \tag{8}$$

Where *q* is elementary charge, *k* is Boltzmann's constant, *T* is temperature, ω is the angular frequency. The V_{bi} is the build-in potential, which is derived from the Mott-Schottky analysis of the *C*-*V* spectra. The E_ω is the demarcation energy for trap state, which can be calculated using the following equation:

$$E_\omega = kTln\left(\frac{\omega_0}{\omega}\right) \tag{9}$$

Where ω_0 is the attempt-to-escape angular frequency, which is assumed to be 10^{12} Hz.

Defects density (N_{CV} and N_{DLCP})

The defects density (N_{CV}) can be calculated from C-V spectra by using following equation:

$$N_{\mathrm{CV}} = -\frac{2}{q\varepsilon_0\varepsilon_{\mathrm{r}}A^2}\left(\frac{dV}{dC^{-2}}\right) \tag{10}$$

There is a nonlinear relationship between the change of charges and the AC bias

$$\frac{\delta Q}{\delta V} = C_0 + C_1\delta V + C_2(\delta V)^2 + C_3(\delta V)^3 + \cdots \tag{11}$$

By the polynomial fitting, C_0 and C_1 were obtained. Then the carrier density (N), which includes free carrier density (N_0) and trap density (N_{DLCP}), at position $X_d = \varepsilon_r\varepsilon_0 A/C_0$ from the junction barrier can be calculated from following equation:

$$N = N_0 + N_{\mathrm{DLCP}} = -\frac{{C_0}^3}{2q\varepsilon_0\varepsilon_{\mathrm{r}}A^2C_1} \tag{12}$$

The N_0 can be estimated by the carrier density measured at a high AC frequency (where the total carrier density tend to saturate with increasing AC frequency). Then the N_{DLCP} can be estimated by $N_{DLCP} = N - N_0$ at a lower AC frequency.

Responsivity (R) and specific detectivity (D^*)

The R is calculated by following equation:

$$R = \frac{EQE \times q\lambda}{hc} \tag{13}$$

where λ is wavelength, h is the Planck constant, and c is the speed of light.

The D^* is calculated by following equation:

$$D^* = \frac{\sqrt{A\Delta f}}{\mathrm{NEP}} = \frac{R\sqrt{A\Delta f}}{i_{\mathrm{n,rms}}} = \frac{R\sqrt{A\Delta f}}{\sqrt{\int_{f_{high}}^{f_{low}} {S_n}^2 df}} \tag{14}$$

where A is the effective device area, Δf is the electrical bandwidth (1 Hz), flow and fhigh are the lowest measured signal frequency and the highest measured signal frequency, respectively.

Modulation transfer function (MTF) analysis

Edge detection is performed using the Sobel operator. The edge spread function (ESF) is then extracted in both the horizontal and vertical directions. Taking the first derivative of the ESF yields the line spread function (LSF), from which the modulation transfer function (MTF) is

calculated via FFT. The spatial frequency at which the MTF drops to 0.5 is defined as the MTF50 value.

2. Notes

Discussion of the potential interface-related effects at MoO_x /Ag interface

Influence of interface localized states: MoO_x thin films fabricated via thermal evaporation contain surface defects and dangling bonds, which give rise to the formation of surface trap states. This observation has been validated in this work and corroborated by previous literature [74,75]. Abundant interface localized states tend to induce a Fermi level pinning effect, which further leads to charge screening behavior, manifested as a weakened response of device current to the applied bias. In the experimental measurements, an enhanced current response to the applied bias was observed after the insertion of the HIES layer; this phenomenon is consistent with the passivation effect of the HIES layer on interface localized states. The mechanism underlying the suppressed electron injection caused by the reduction of interface localized states has been discussed in the main text. However, this mechanism fails to explain a key contradiction: under the same reverse bias, $BArF4^-$-based devices (which exhibit superior passivation performance) show a higher dark current density than Br^--based devices.

Influence of image charge effects: For the MoO_x/electrode metal-semiconductor contact, its contact type can be deduced from the Fermi level alignment between MoO_x and the electrode. This contact is of an ohmic type rather than a Schottky type. In the context of image charge effects, the image force acts in the direction from the metal to the n-type semiconductor, which reduces the actual height of the Schottky barrier relative to its ideal value. Notably, this effect exerts no significant influence on ohmic contacts. On this basis, it is concluded that the experimental phenomena observed in this work are unrelated to image charge effects.

Influence of interface dipoles: Previous literature has confirmed that the modification of WF by CPEs is primarily attributed to the generation of interface dipoles [76,77]. Accordingly, the changes in WF observed in the this work reflect the macroscopic manifestation of interface dipole effects. However, as noted in the main text, a theoretical model relying solely on interface dipoles or WF cannot fully account for the experimental phenomena. Therefore, interface dipoles are not considered the primary cause of the observed effects in this study.

3. Figures and Tables

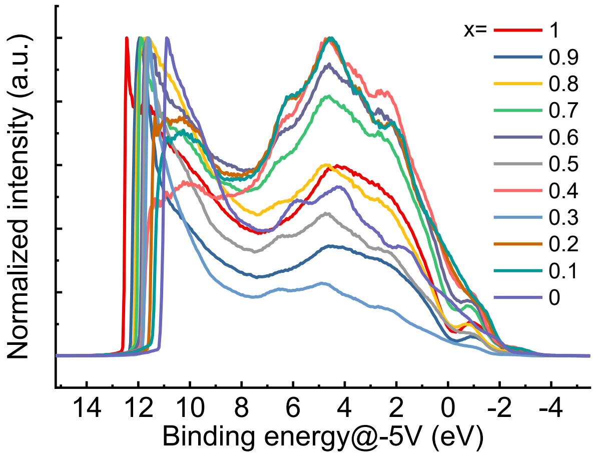

Figure S1. The raw data obtained from UPS of Ag electrode modified by HIES with different Br^- proportion x.

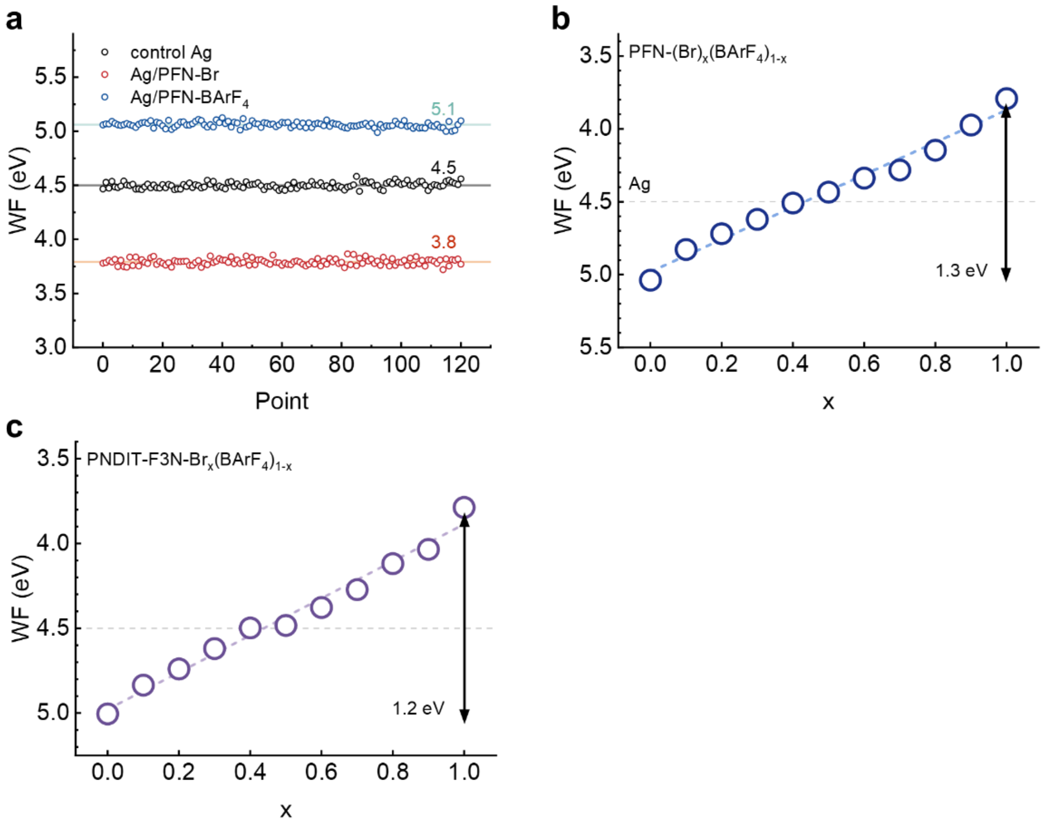

Figure S2. (**a**) The WF of Ag modified by PFN-Br and PFN-BArF$_4$, measured by KPFM. To exclude interference, 120 points per sample were tested and averaged as a result. (**b-c**) The linear change in WF of Ag modified by PFN-Br$_x$(BArF$_4$)$_{1-x}$ (**b**) and PNDIT-F3N-Br$_x$(BArF$_4$)$_{1-x}$ (**c**).

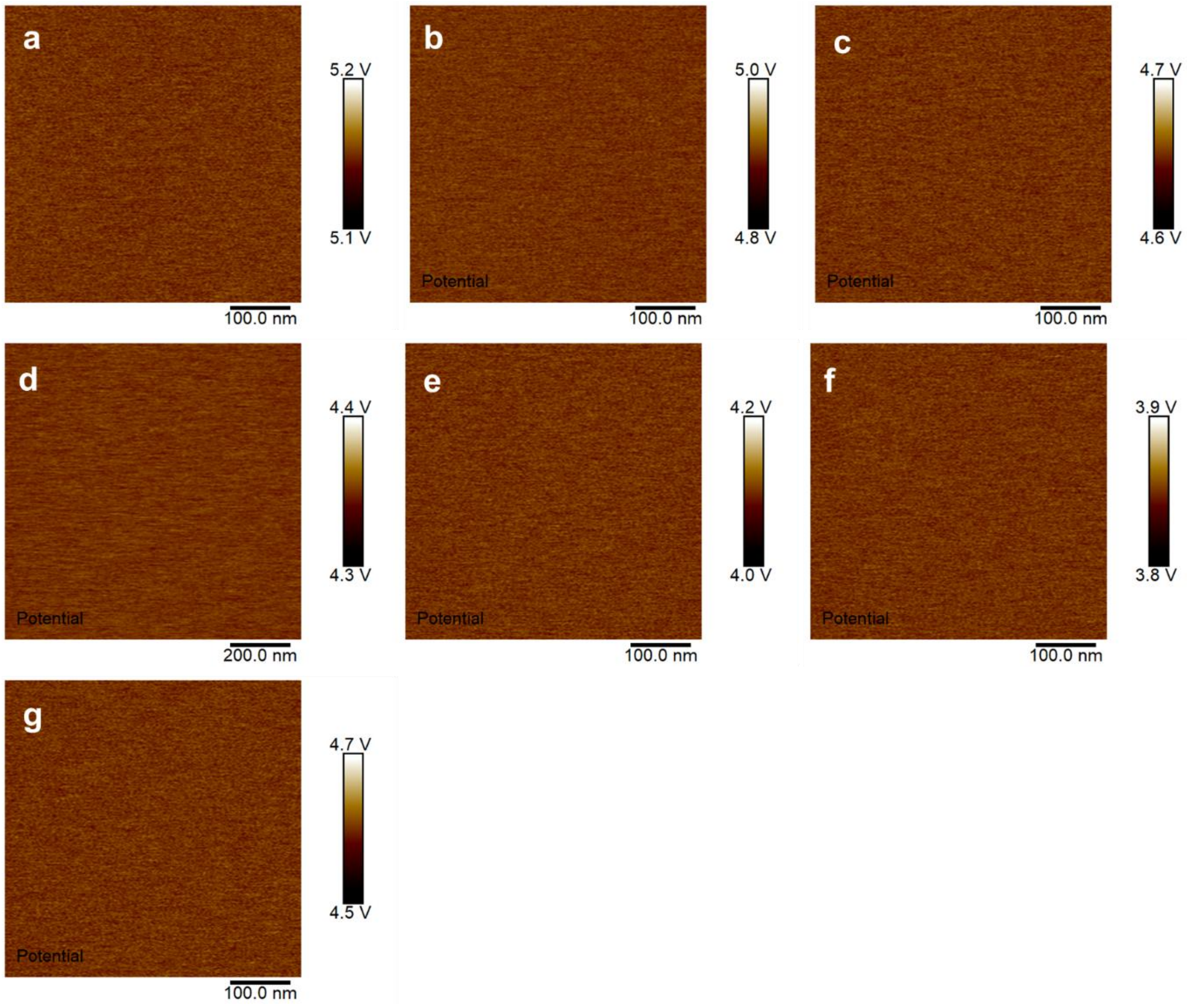

Figure S3. (**a-f**) The KPFM of Ag modified by PFN-$Br_x(BArF_4)_{1-x}$ with varying Br^- proportion x: (**a**) x=0, (**b**) x=0.2, (**c**) x=0.4, (**d**) x=0.6, (**e**) x=0.8 and (**f**) x=1. **g,** KPFM of a highly oriented pyrolytic graphite (HOPG) reference sample, with a WF of 4.6 eV.

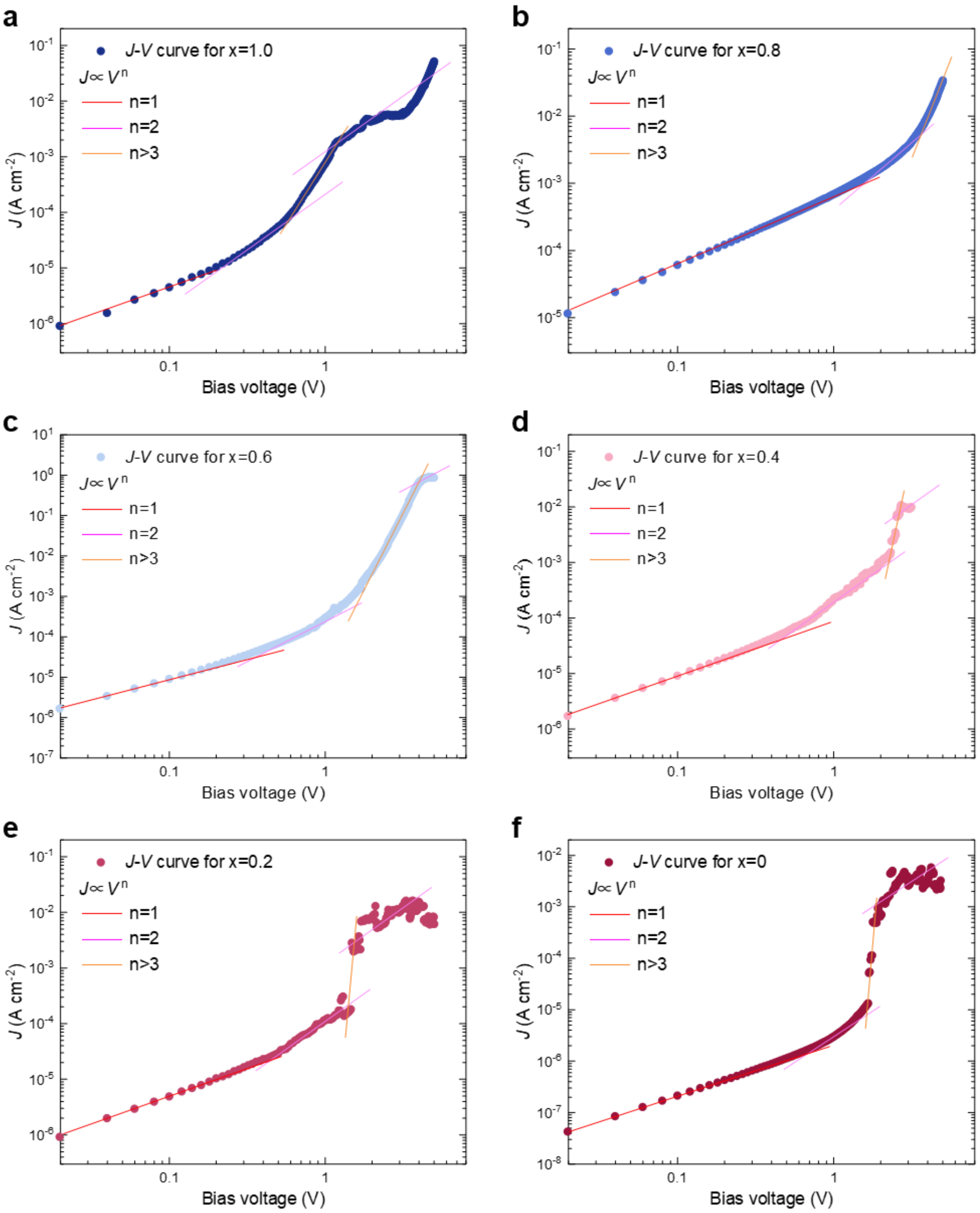

Figure S4. The *J-V* curves of electron-only devices. The thick HIES layers (~80 nm - 105 nm) with different Br^- ratios (**a**) x = 1, (**b**) x = 0.8, (**c**) x = 0.6, (**d**) x = 0.4, (**e**) x = 0.2 and (**f**) x = 0 were deposited between ZnO layer and C_{60} layer. The first region with a slope of n = 2 in the *J-V* curves corresponds to the intrinsic SCLC region (traps in the film remain unfilled), where data from this region were used to calculate the intrinsic mobility of HIES.

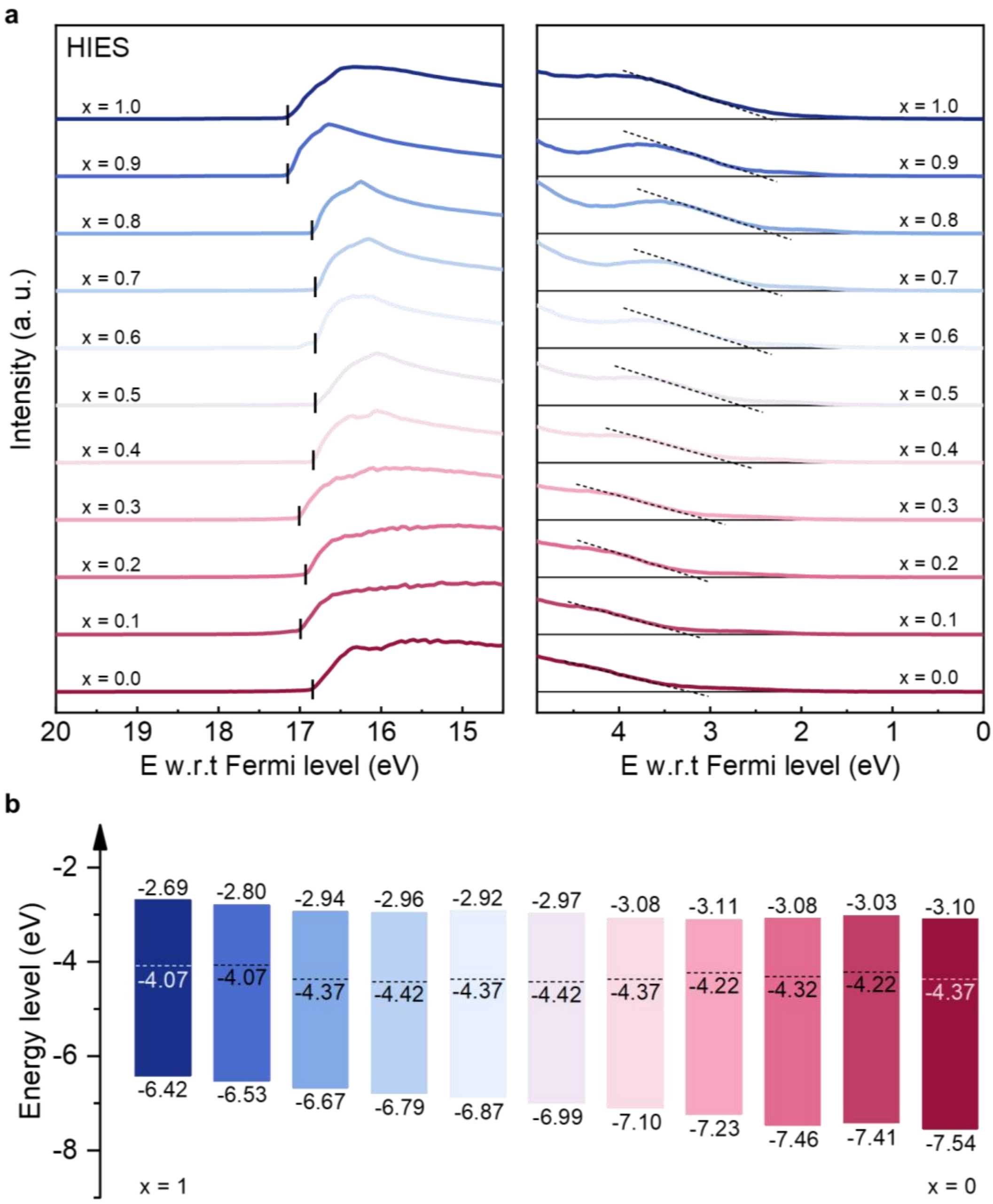

Figure S5. (**a**) The UPS spectra of the PFN-$Br_x(BArF_4)_{1-x}$ films. (**b**) Energy levels diagram of the PFN-$Br_x(BArF_4)_{1-x}$ films.

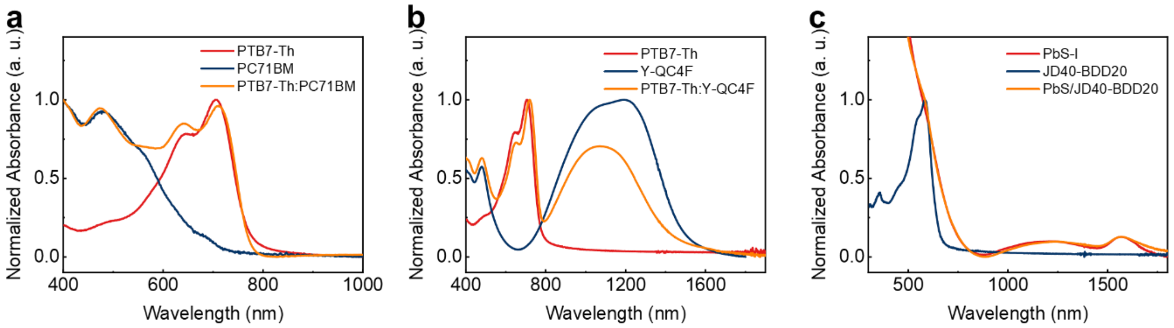

Figure S6. The absorption spectra of (**a**) PTB7-Th:PC_{71}BM, (**b**) PTB7-Th:Y-QC4F and (**c**) PbS/JD40-BDD20 films. Quartz substrates were employed, and the instrument was calibrated against blank quartz slides before measurements were taken. Each figure also displays the absorption spectra of the corresponding single-component films to facilitate analysis of charge-transfer (CT) state absorption. No distinct CT state absorption features were observed in any of the three systems.

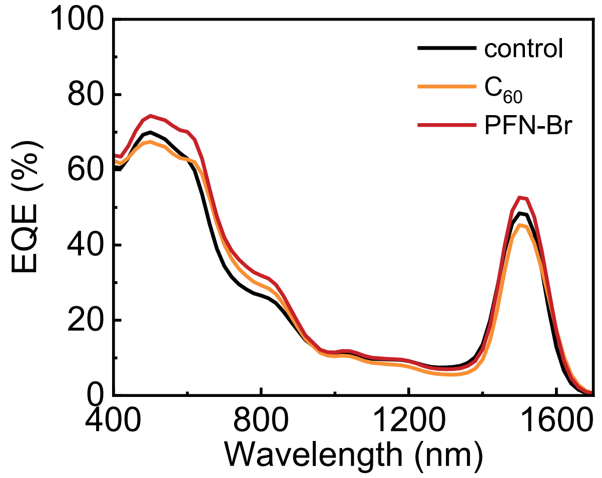

Figure S7. The EQE-wavelength curves of devices with MoO_x/Ag (control), MoO_x/C_{60}/Ag (C_{60}) or MoO_x/PFN-Br/Ag (PFN-Br) structure.

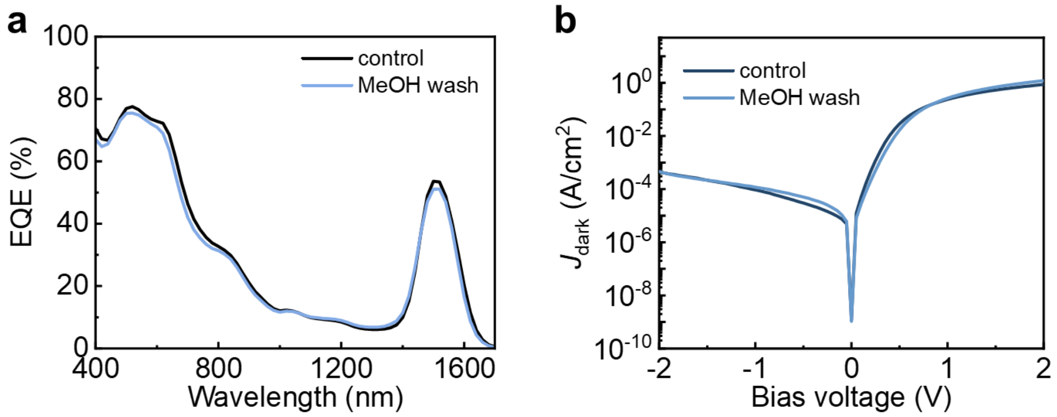

Figure S8. The (**a**) EQE-wavelength and (**b**) J_{dark}-V curves of devices with and without MeOH wash. The minimal performance variation indicates that solution processing with MeOH as the solvent neither disrupts the MoO_x structure nor degrades device performance.

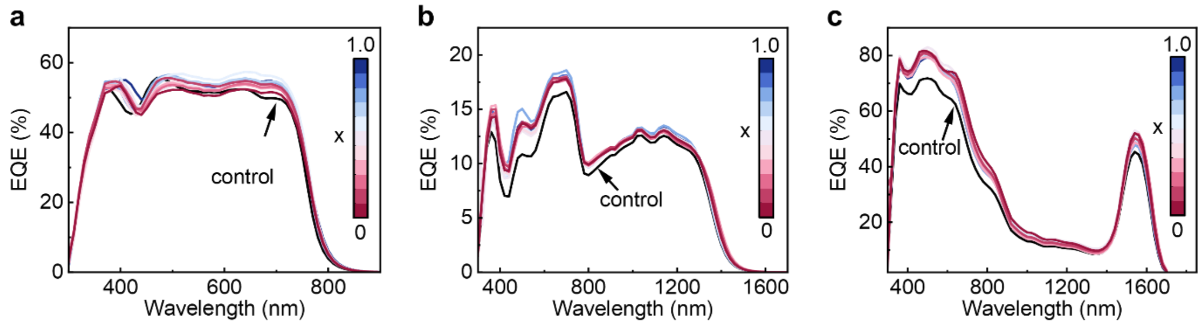

Figure S9. The EQE-wavelength curves of the PDs with PFN-$Br_x(BarF_4)_{1-x}$, whose photosensitive layers were (**a**) PTB7-Th:$PC_{71}BM$, (**b**) PTB7-Th:Y-QC4F and (**c**) PbS CQDs/JD40-BDD20. The black lines belong to the control devices.

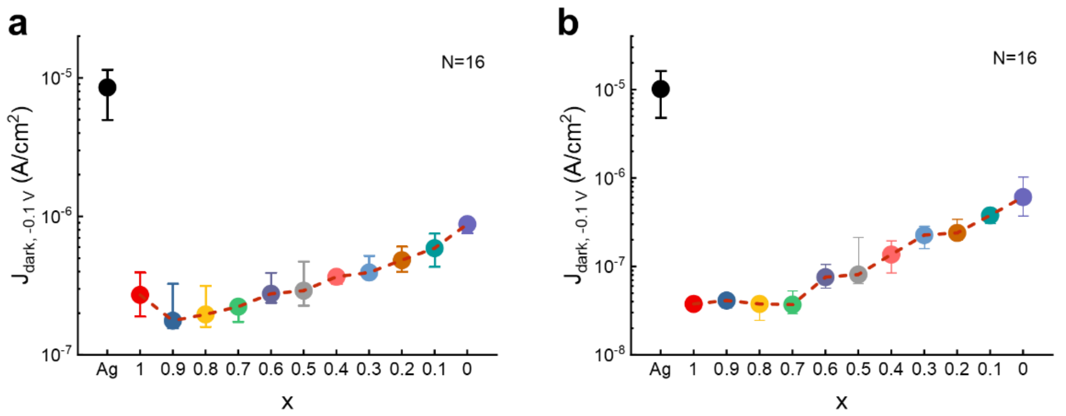

Figure S10. (**a** and **b**) Statistical analysis of the dark current J_{dark} at −0.1 V reverse bias, based on independent repetitions across devices. N represents the sample size for each experimental group.

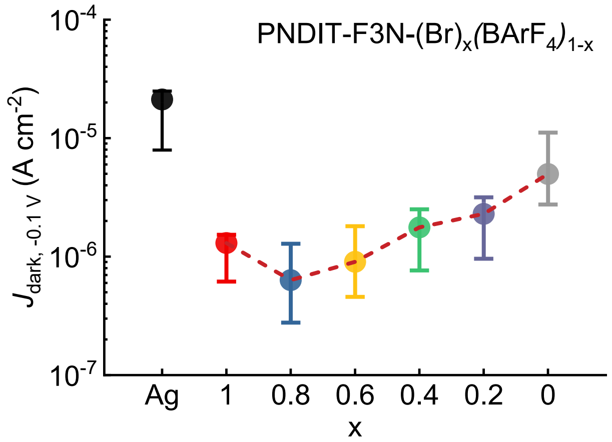

Figure S11. Statistical analysis of J_{dark} at −0.1 V reverse bias across devices involving PNDIT-F3N-$(Br)_x(BArF_4)_{1-x}$. The sample size of each experimental group is 16.

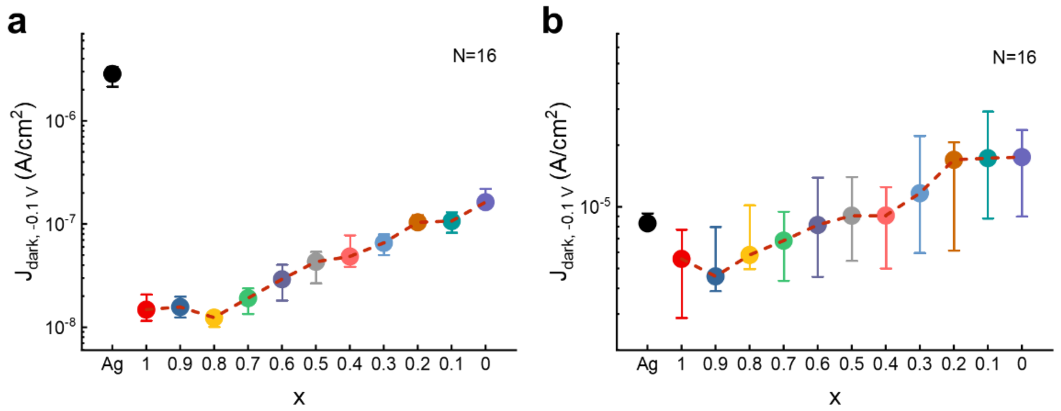

Figure S12. Statistical analysis of J_{dark} at −0.1 V reverse bias across devices based on (**a**) PTB7-Th:IEICO-4F and (**b**) P1:Y7, respectively. N is the sample size of each experimental group.

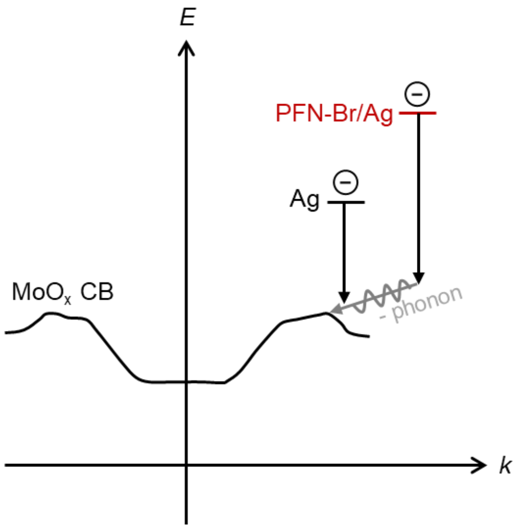

Figure S13. Schematic diagram of the energy bands and electron transition processes in momentum space when the HIES-modified Ag electrode is in contact with MoO_x. According to the free-electron model, the energy (E) and momentum (p) of electrons at the Fermi level of Ag satisfy the relationship: $E_{Fermi} = p^2/(2m_e) = (\hbar k)^2/(2m_e)$, where a higher E_{Fermi} corresponds to greater p and wavevector (k). Electrons tend to transition to the highest point in the CB of MoO_x in momentum space. If momentum is mismatched, phonons participate to conserve momentum. It should be noted that in complex amorphous thin film systems, the free-electron model cannot be used for a precise quantitative description of the system's state. However, the trend relationship it reveals between the WF and the momentum of free electrons within metals remains valuable.

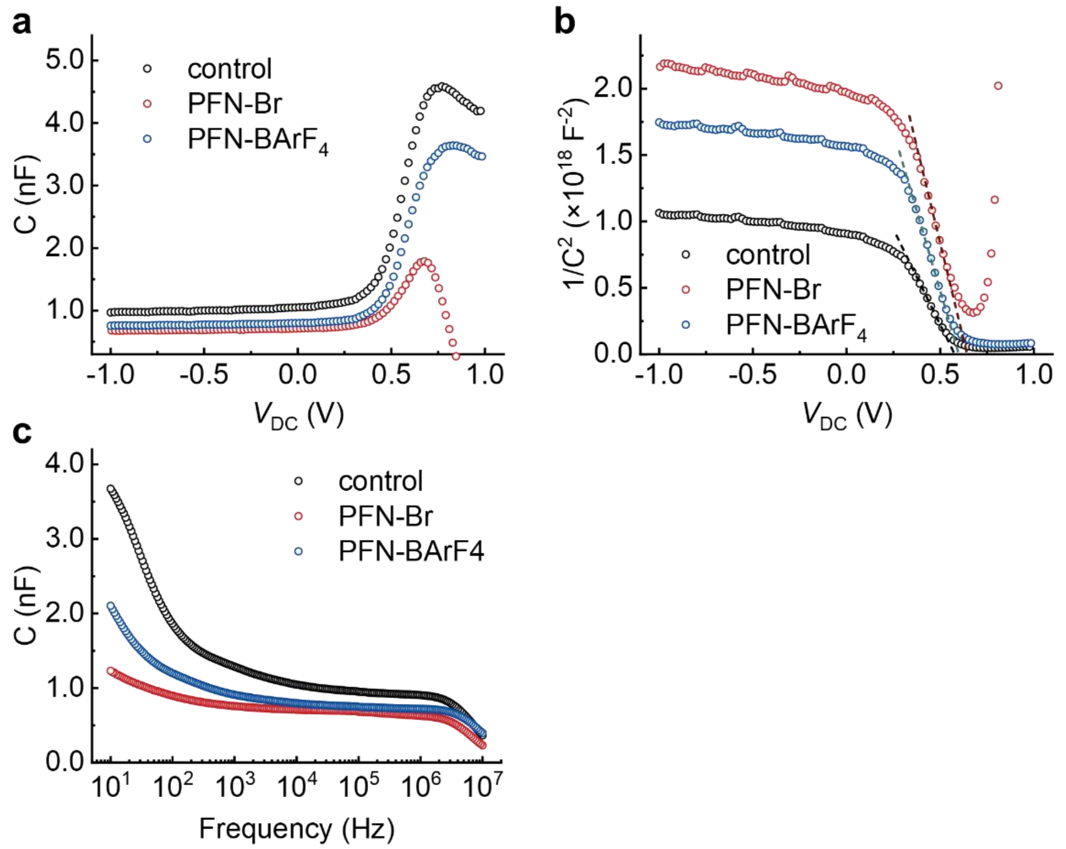

Figure S14. (**a**) *C*–*V* characteristics, (**b**) Mott–Shockley plots (dashed lines represent the linear fits) and (**c**) *C*–*f* curves for the devices with and without HIES layer.

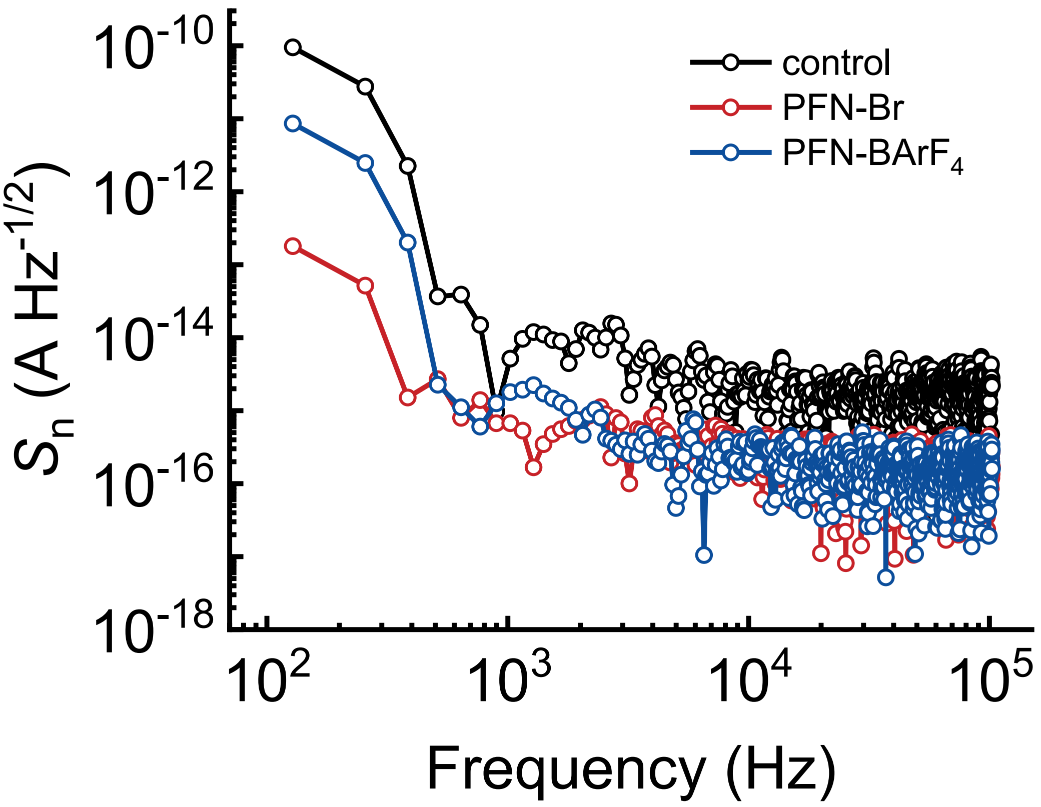

Figure S15. The spectral density of noise current (S_n) of devices with and without HIES layer, measured at 0 V bias, with thermal noise as the primary contributor.

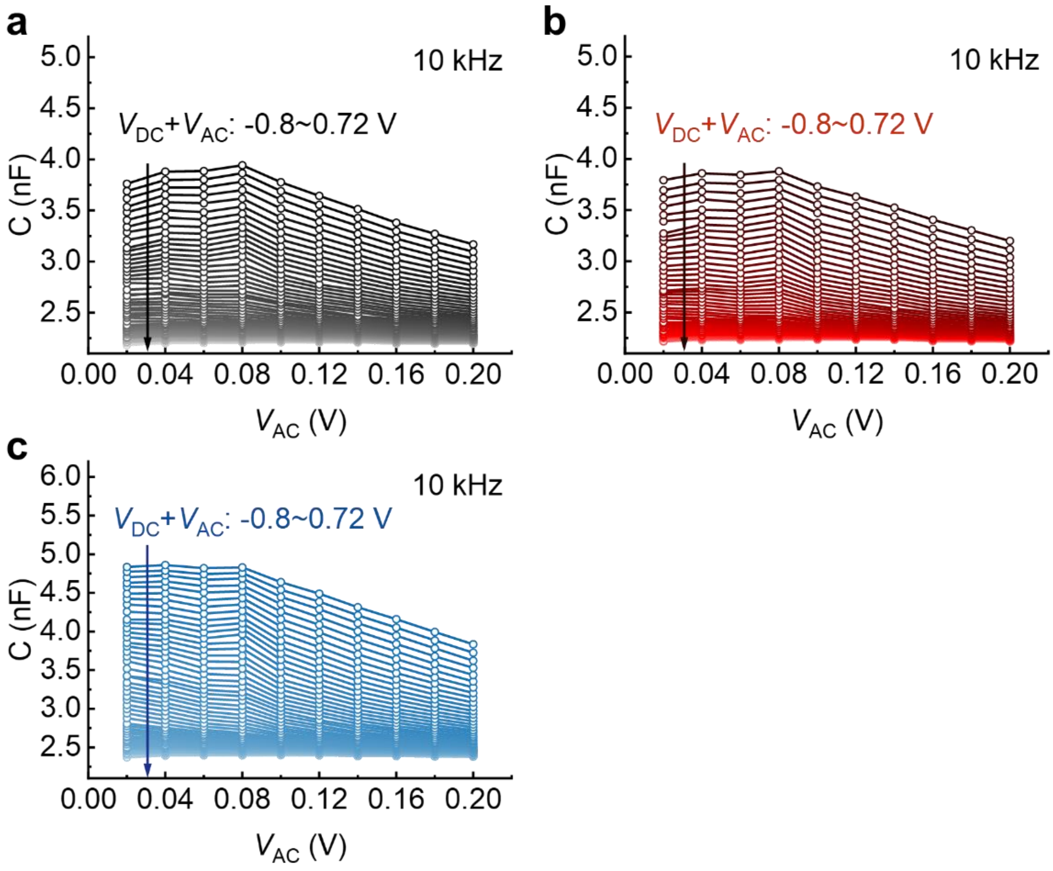

Figure S16. The V_{AC} dependent capacitance of devices: (**a**) without HIES layer and (**b**) with PFN-Br and (**c**) with PFN-BArF$_4$. The $V_{DC}+V_{AC}$ is ranging from −0.8 to 0.72 V with a 20 mV step.

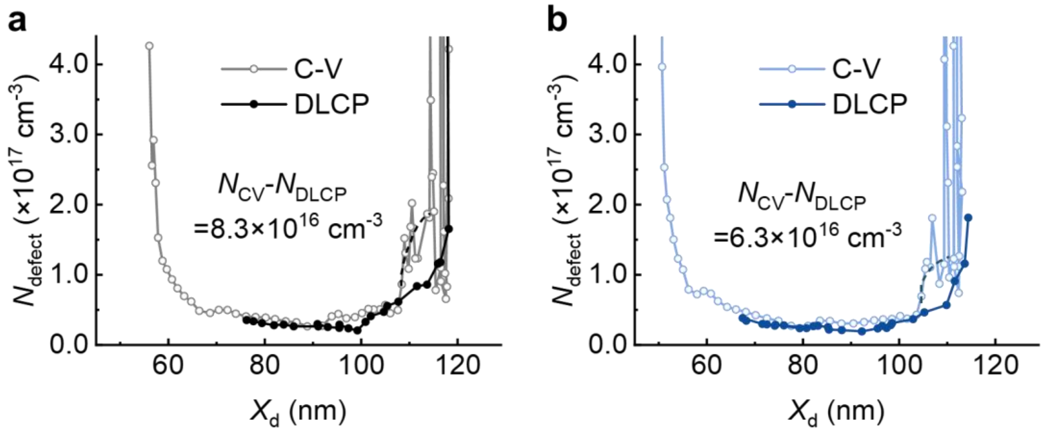

Figure S17. Defect density profiles of (**a**) control devices and (**b**) devices with PFN-BArF$_4$. The defect densities were measured by *C*-*V* and DLCP. The difference between the N_{CV} and N_{DLCP} represents the defect density originating from the interface.

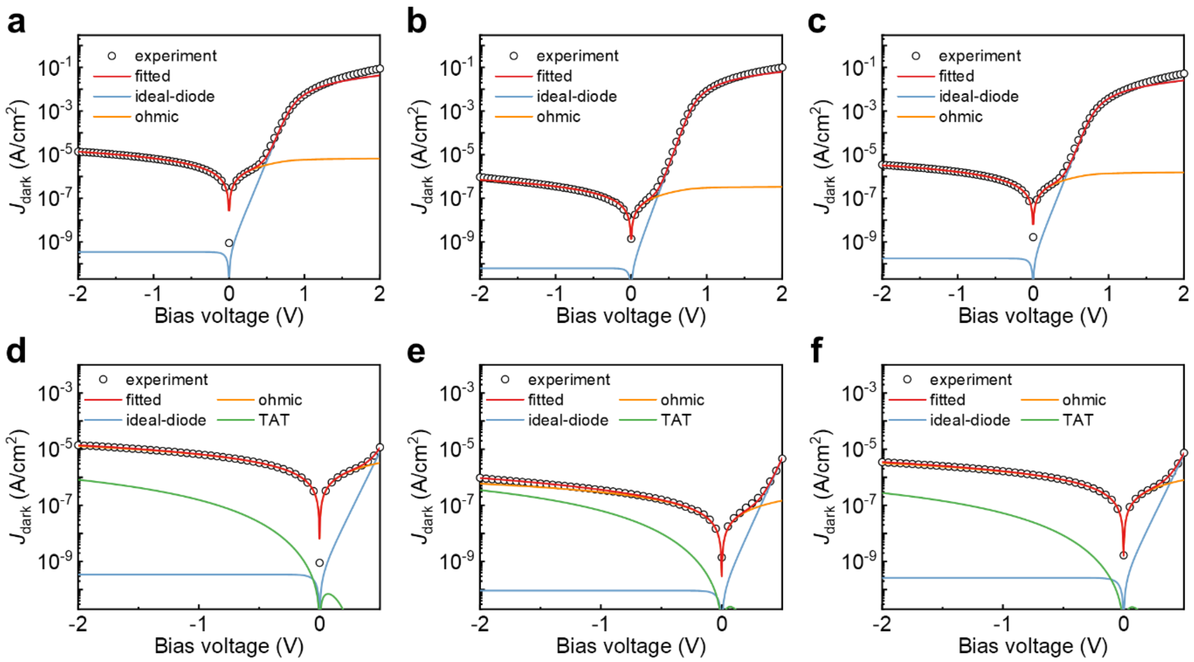

Figure S18. The experimental and fitted J_{dark}-V curves of (**a,d**) control devices, the devices with (**b,e**) PFN-Br or (**c,f**) $BArF_4$. (**a-c**) show the results of preliminary fitting using the first two terms of the equation, and (**d-f**) present the results of fitting with the complete equation.

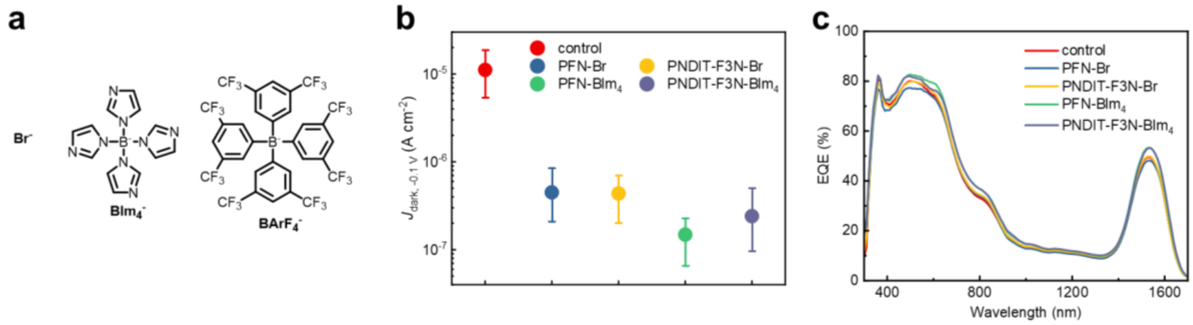

Figure S19. (**a**) The structures of the three counterions. (**b**) Comparison of J_{dark} for different devices under −0.1 V bias. (**c**) Comparison of EQE for devices with different HIES.

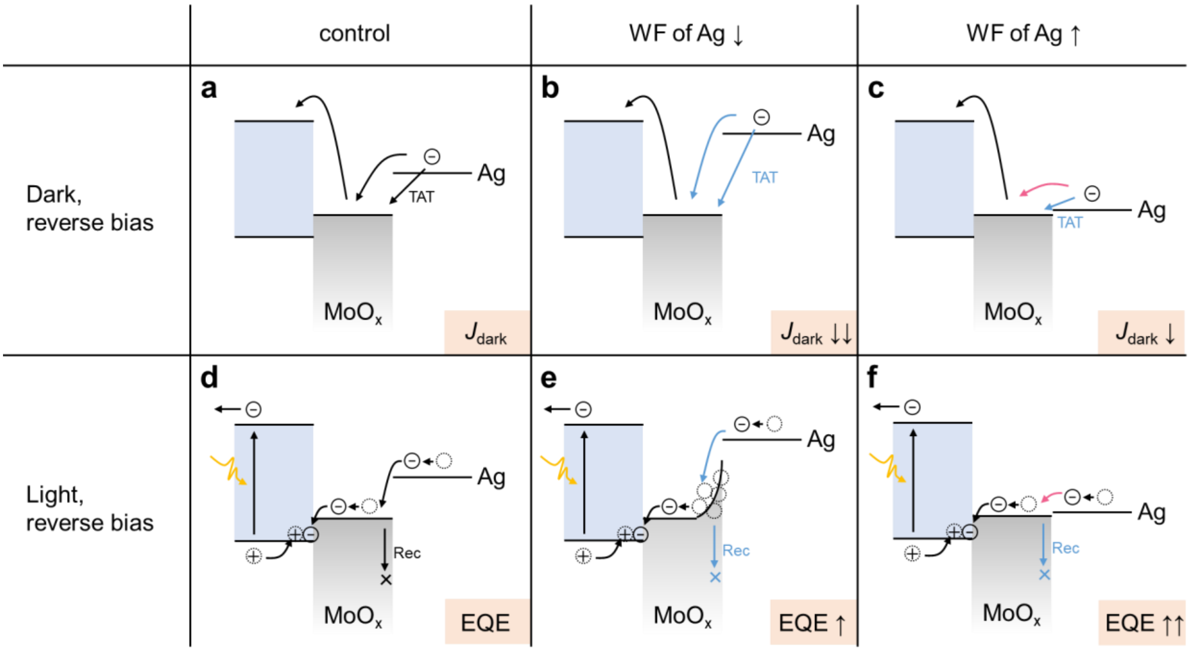

*Blue arraos (⟶) indicate that the process is inhibited; red arrows (⟶) indicate that the process is promoted.

Figure S20. Schematic diagram for analyzing the mechanism of HIES action. Based on the experimental results (**Fig. 4f**), it was confirmed that HIES modification alters the electron injection barrier from the electrode into the device. Specifically, when the Fermi level (E_{Fermi}) of Ag is shifted farther from the conduction band (CB) of MoO_x by HIES modification, electron injection becomes more difficult; conversely, when E_{Fermi} is brought closer to the CB of MoO_x, electron injection is facilitated. In addition, HIES passivates interface defects and reduces the density of interfacial trap states. It is also established that MoO_x exhibits a recombination-type hole-extraction mechanism, wherein photogenerated holes from the HOMO of the photosensitive layer recombine with electrons from dopant levels near the MoO_x CB, thereby enabling effective hole extraction. Based on these three considerations, analysis of carrier transport under both dark and illuminated conditions as follows: (**a**) Under reverse bias in the dark condition, electrons are injected from the Ag electrode into the CB of MoO_x, and then transition to the LUMO of the photosensitive layer, completing the injection process. (**b**) When E_{Fermi} of Ag is shifted away from the MoO_x CB by HIES, electron injection becomes more difficult from an energetic perspective, and the reduced trap state density suppresses trap-assisted tunneling, leading to a reduction in J_{dark}. (**c**) If HIES brings E_{Fermi} closer to the CB, injection becomes energetically more favorable, but the dominant effect of suppressed trap-assisted tunneling still results in a decrease in J_{dark}, albeit to a lesser extent. (**d**) Under reverse bias under illumination, photogenerated holes from the HOMO of the photosensitive layer recombine with electrons from dopant levels near the MoO_x CB, achieving effective hole collection into MoO_x, while electrons from the Ag electrode refill into MoO_x, completing the

hole extraction process. (**e**) If HIES shifts E_{Fermi} of Ag away from the MoO_x CB, initial electron refilling from Ag becomes more difficult. This causes hole accumulation near the MoO_x/Ag interface, resulting in upward band bending of MoO_x, which in turn reduces the energy barrier between the MoO_x CB and E_{Fermi} of Ag, facilitating subsequent electron refilling. Simultaneously, the lower trap state density suppresses trap-assisted recombination. Together, these effects enhance the EQE, though at the cost of a slightly slower photoresponse, as observed in **Fig. S27**. (**f**) Alternatively, when E_{Fermi} is brought closer to the MoO_x CB, electron refilling from Ag becomes more efficient, and the reduction in trap-assisted recombination likewise contributes to improved EQE.

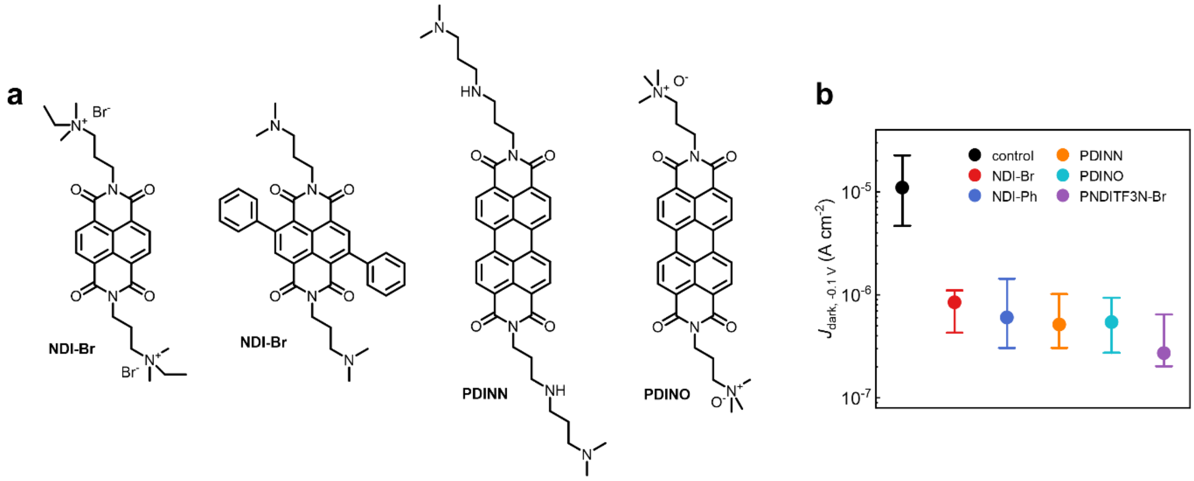

Figure S21. (**a**) The structures of the small-molecule HIES. NDI-Br and NDI-Ph have a similar unit structure to PNDIT-F3N-Br. (**b**)Statistical analysis of J_{dark} at −0.1 V reverse bias across devices based on small-molecule HIES.

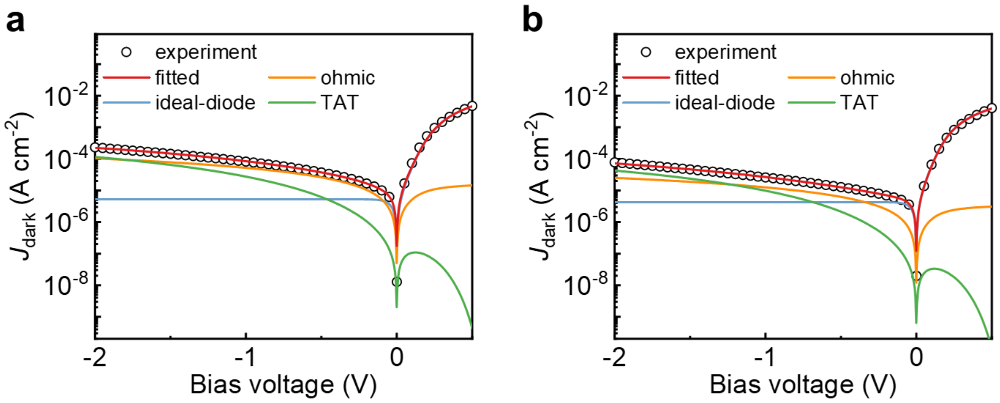

Figure S22. The experimental and fitted J_{dark}-V curves of devices (**a**) without and (**b**) with HIES layer. The photosensitive layer is PTB7-Th:Y-QC4F.

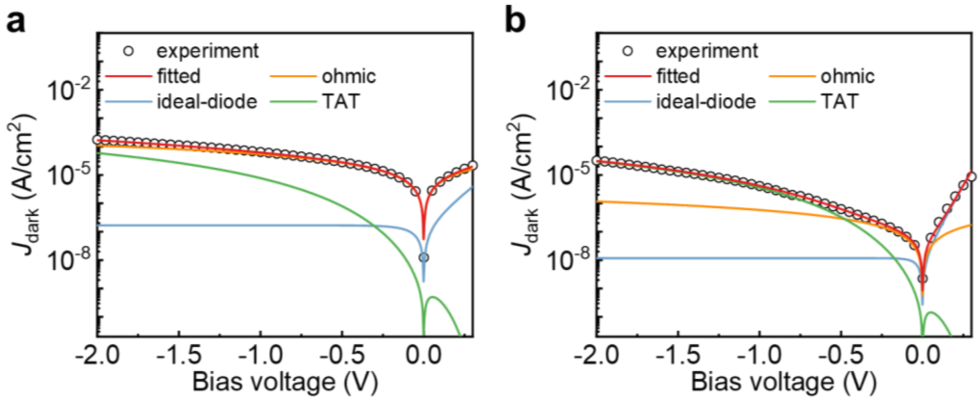

Figure S23. The experimental and fitted J_{dark}-V curves of (**a**) control devices and (**b**) the optimized devices with HIES layer. The photosensitive layer is PbS/JD40-BDD20.

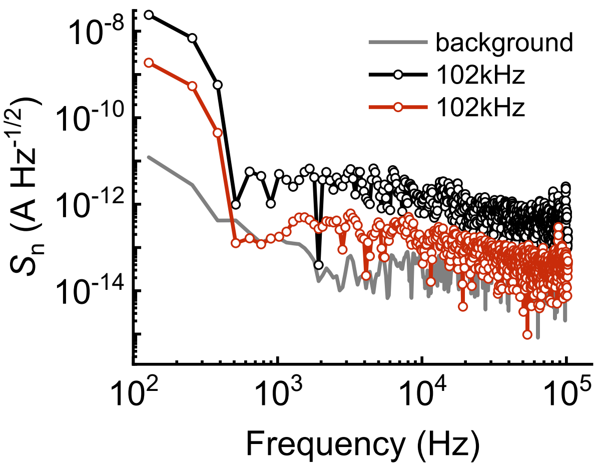

Figure S24. The S_n of devices with and without HIES layer measured in range of 128 Hz to102.4 kHz under −0.1 V bias.

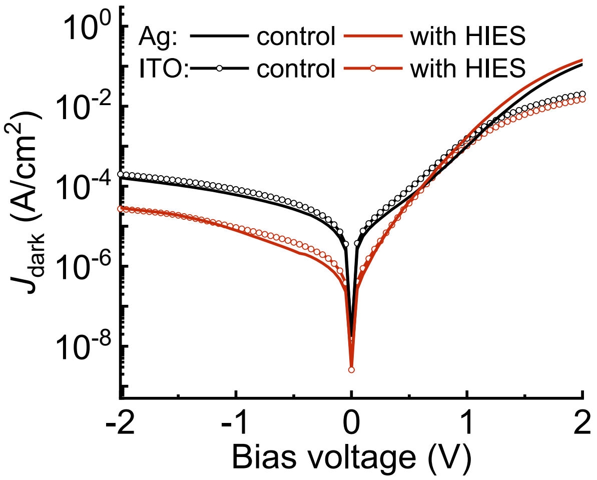

Figure S25. The J_{dark}-V curves for devices with different top electrodes.

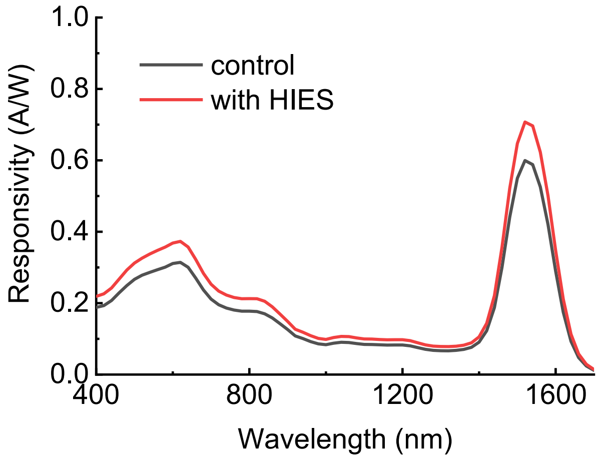

Figure S26. The responsivity (R) of devices with and without HIES layer, measured under 0 V bias.

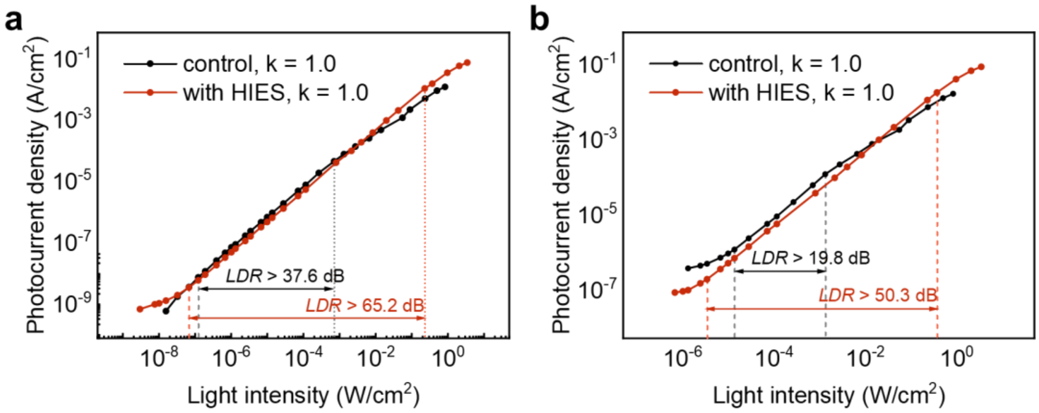

Figure S27. The linear dynamic range (*LDR*) of devices with and without HIES layer, measured under (**a**) 0 V and (**b**) −0.1 V bias. The *LDR* is calculated by equation (5).

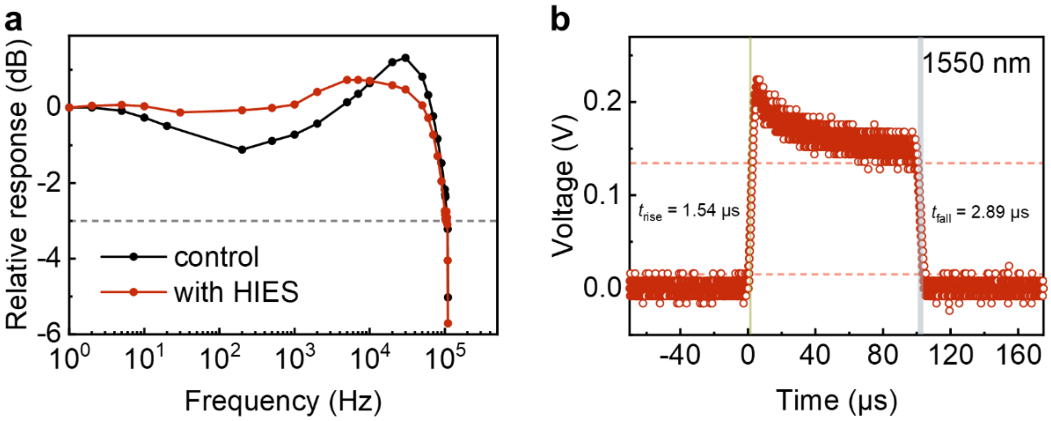

Figure S28. (**a**) The response bandwidth and (b) response speed of devices. In the measurement of response speed, the light source was modulated with a square wave signal at 10 kHz.

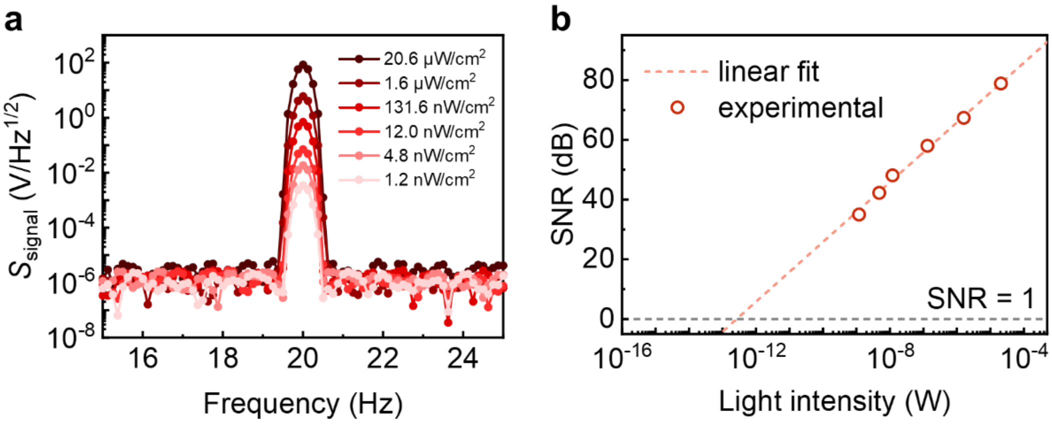

Figure S29. Noise equivalent power (NEP) measurements under 1550 nm light source. (**a**) Photoresponse at a single modulation frequency (20 Hz, 1 Hz bandwidth) under varying light intensities. (**b**) Estimation of the NEP by extrapolating the light intensity-dependent SNR to unity. The NEP at low frequency (20 Hz) is demonstrated.

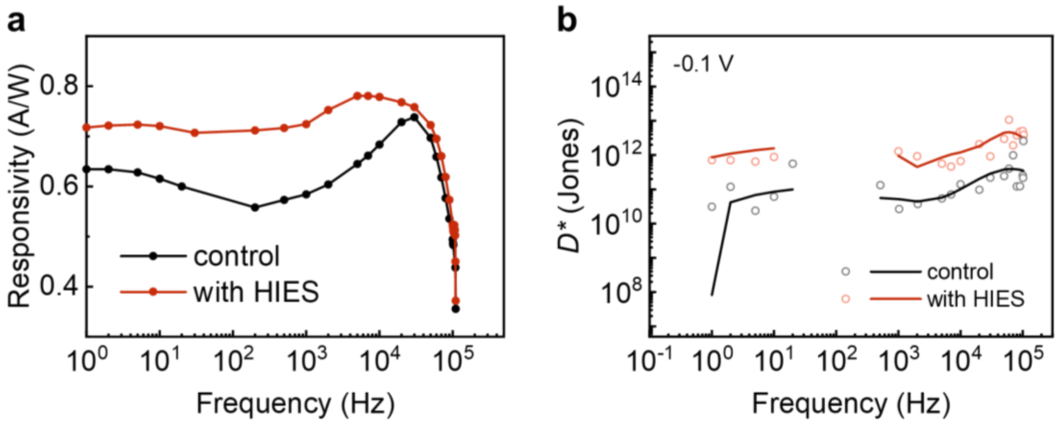

Figure S30. (**a** and **b**) The frequency-dependent responsivity (*R*) and specific detectivity (D^*) of devices, respectively. D^* is calculated from the *R* and the noise power density at the corresponding frequency. In (**b**), the dots represent the D^* derived from the noise power density calculations at the specific frequencies, while the lines show the average of ten data points centered around each frequency to reduce distortion caused by external disturbances.

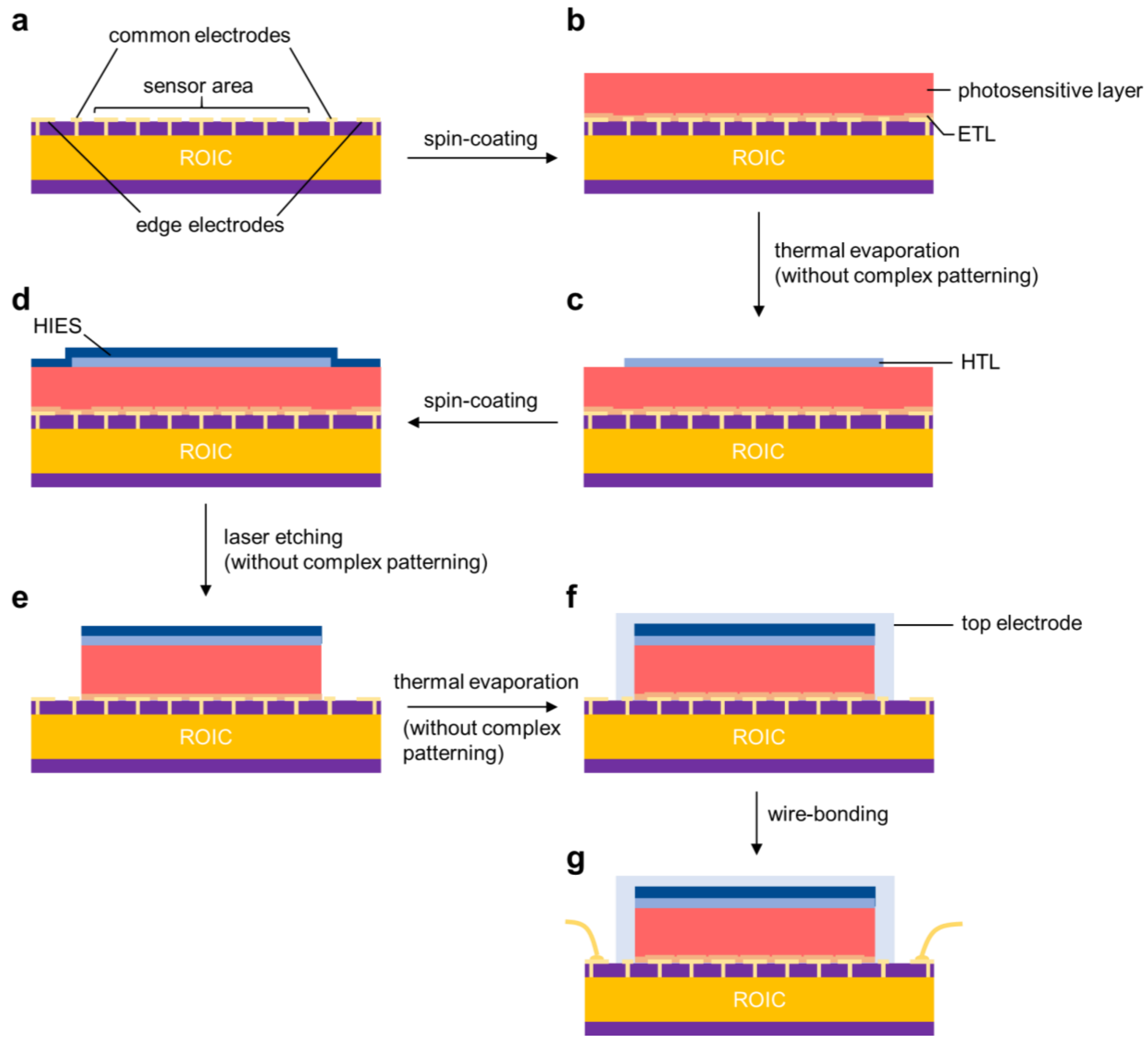

Figure S31. Fabrication process diagram of SWIR FPA imagers. (**a**) the CMOS ROIC. (**b**) ETL layer and photosensitive layer are deposited by spin-coating. (**c**) HTL layer is deposited by thermal evaporation. (**d**) HIES layer is deposited by spin-coating. (**e**) use laser etching to expose the common electrodes and the edge electrodes. (**f**) the top Ag electrode is deposited by thermal evaporation. (**g**) connect to the external circuits by wire-bonding.

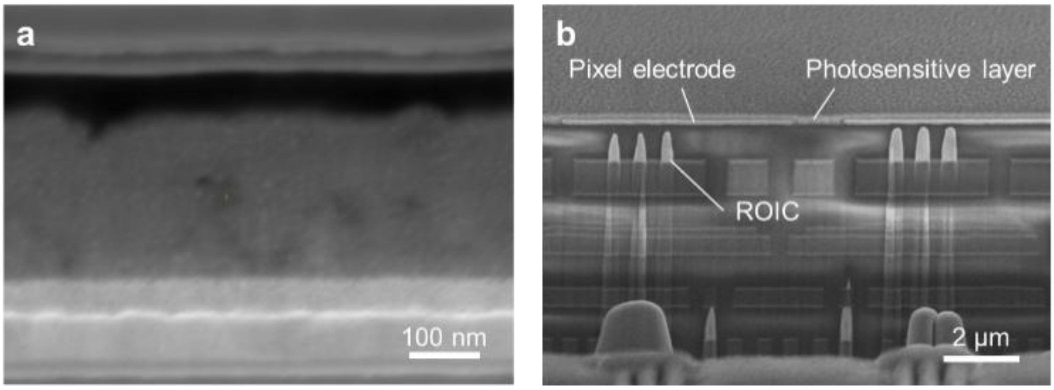

Figure S32. (**a** and **b**) The cross-sectional SEM image of the pixels in SWIR FPA imager, showcasing a continuous, pattern-free photosensitive layer.

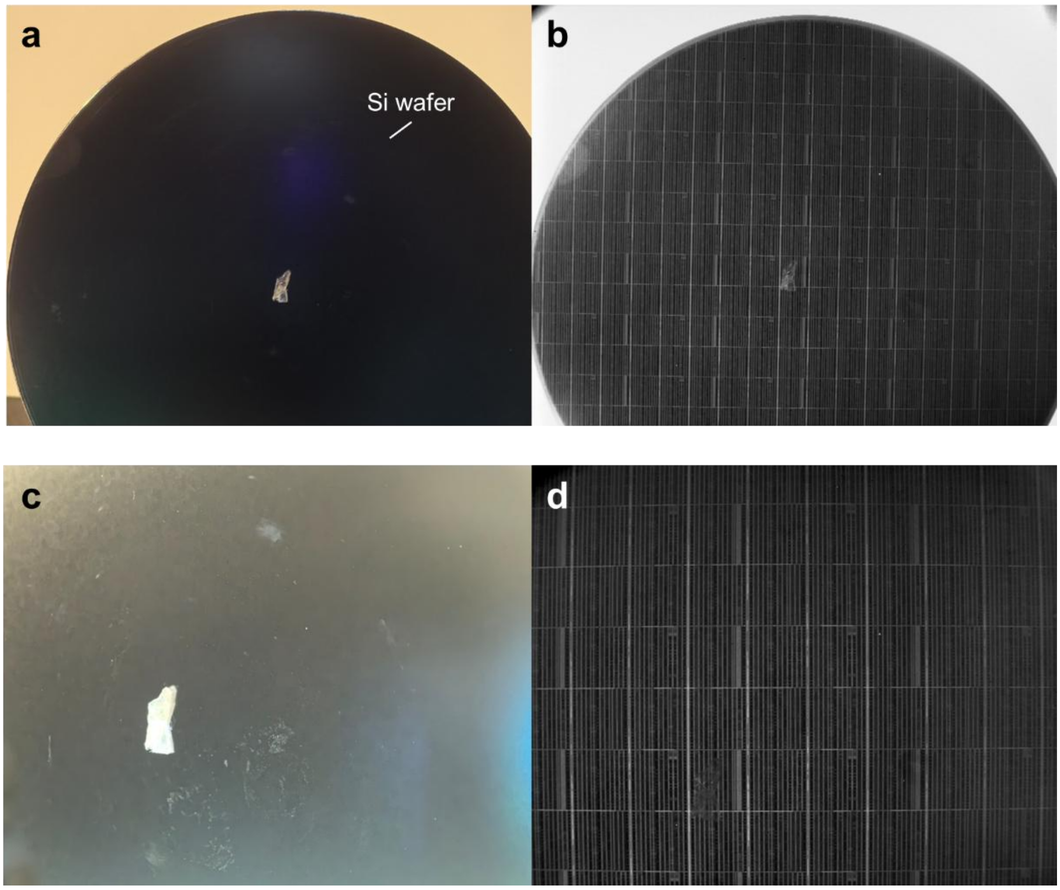

Figure S33. (a-d) Images captured with (**a** and **c**) a commercial visible light camera and (**b** and **d**) our SWIR FPA imaging instrument, demonstrating an application scenario: revealing the internal structure of the chip without damaging the upper layers to detect defects.

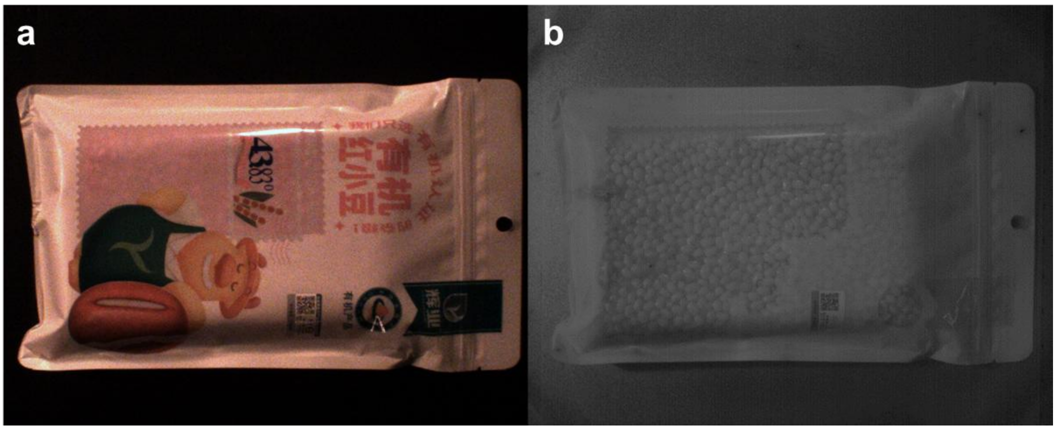

Figure S34. Images captured with (**a**) a commercial visible light camera and (**b**) our SWIR FPA imaging instrument, demonstrating an application scenario: imaging the food inside opaque colored plastic bags without opening them, enabling non-destructive food inspection while preserving the seal.

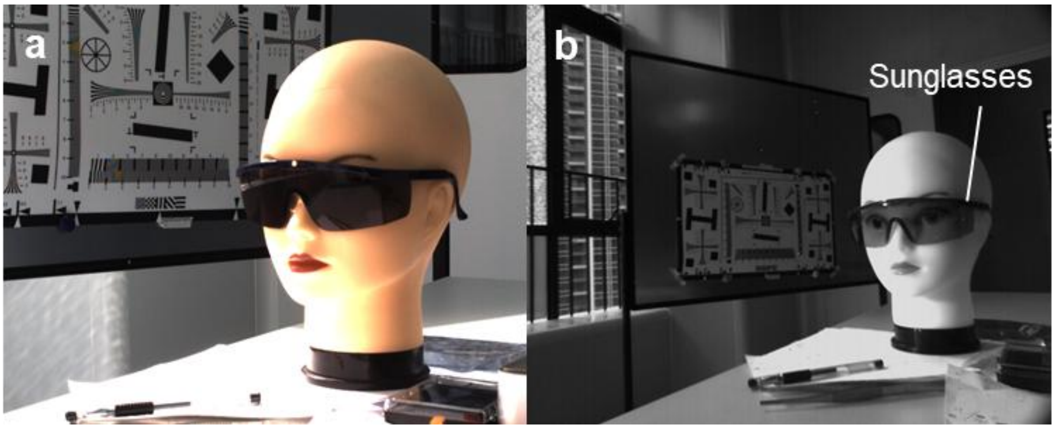

Figure S35. (a and **b)** A facial recognition demonstration of a model wearing sunglasses under natural light using our SWIR FPA imager. The images were captured using (**a**) a commercial visible light camera and (**b**) our SWIR FPA imager at 5 p.m.

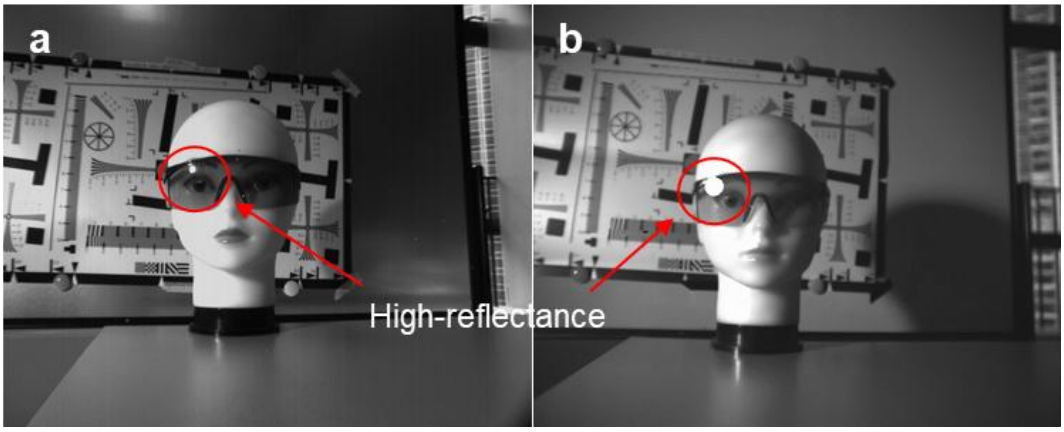

Figure S36. (a and **b)** Comparison of saturation artifacts induced by specular reflection. The images were captured using (**a**) our SWIR FPA imager and (**b**) a commercial InGaAs SWIR imager.

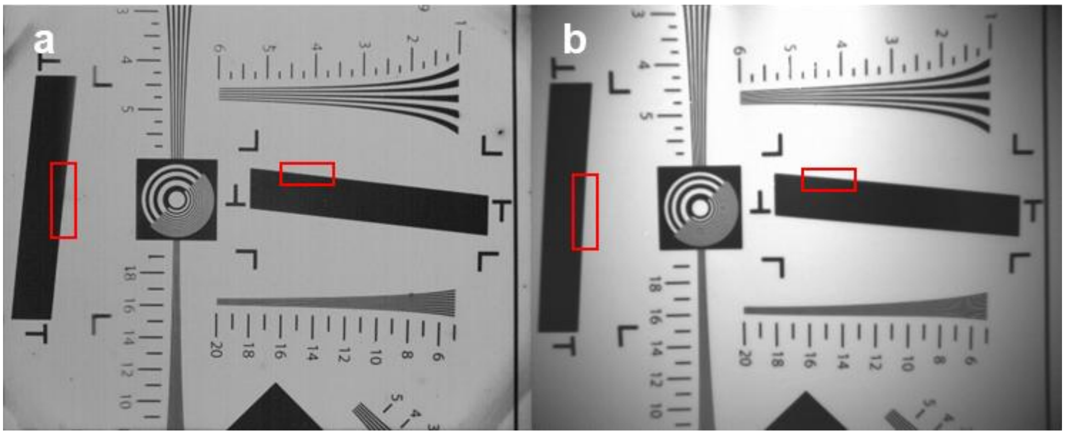

Figure S37. (a and **b)** Comparison of imaging clarity under identical lens parameters (aperture, focal length). The images were captured using (**a**) our SWIR FPA imager and (**b**) a commercial InGaAs SWIR imager. The red-framed area is used for MTF analysis.

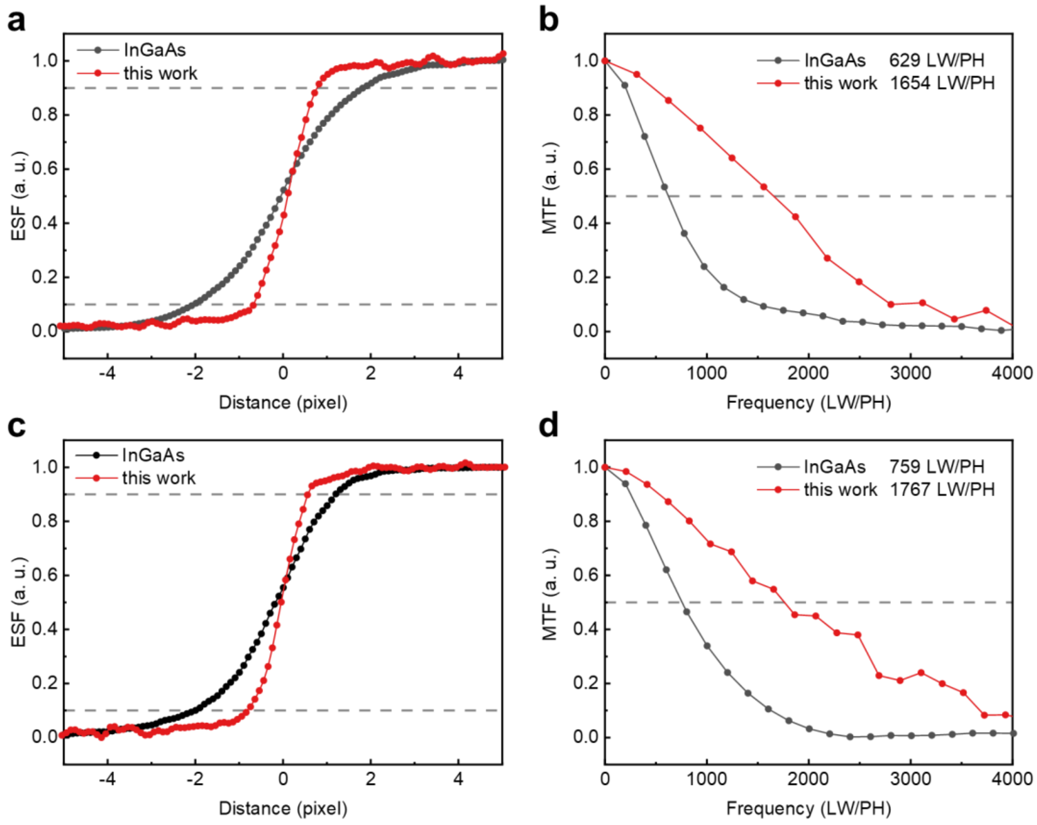

Figure S38. The comparison results of MTF analysis extracted in **(a**,**b)** horizontal and (**c**,**d**) vertical. In (**a** and **c**), our SWIR imager exhibits significantly smaller rise distances (1.4 pixels horizontally and 1.3 pixels vertically), compared to the commercial InGaAs camera for 3.8 pixels horizontally and 3.1 pixels vertically. In (**b** and **d**), The extracted MTF50 corresponds to the ESF results.

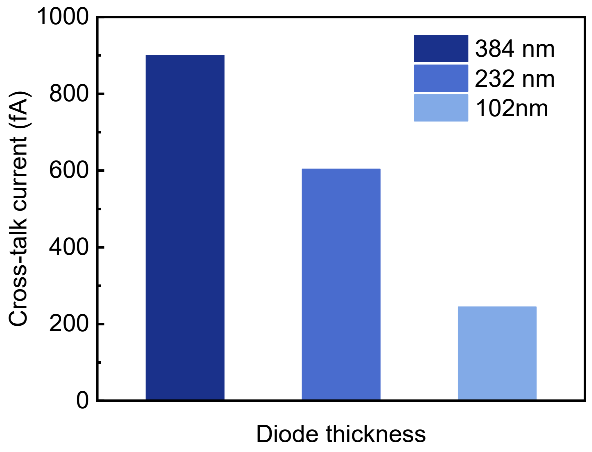

Figure S39. The crosstalk current between adjacent pixels on the dummy chips. The dummy chips consist solely of arrayed pixel electrodes and a solution-processed photodetector layer, without an integrated readout circuit.

Table S1. Work function of Ag/PFN-$Br_x(BArF_4)_{1-x}$ with different Br^- proportion x, measured by KPFM and UPS.

x	Work function [eV]	
	KPFM	UPS
0.0	5.04	5.05
0.1	4.83	4.90
0.2	4.72	4.85
0.3	4.62	4.73
0.4	4.51	4.63
0.5	4.43	4.54
0.6	4.34	4.41
0.7	4.28	4.34
0.8	4.15	4.21
0.9	3.97	4.13
1.0	3.79	4.00

Table S2. The depth, width and density of the trap state (with E_{trap} ranging from 0.35 eV to 0.45 eV) for devices with and without HIES layer were determined. These characteristics were derived from the fitting of Gaussian distribution.

Device	Depth of trap state [eV]	Width of trap state [eV]	N_t [cm^{-3}]
control	0.41	0.06	2.0×10^{14}
with PFN-Br	0.38	0.08	4.0×10^{14}
with PFN-BArF$_4$	0.41	0.05	1.3×10^{14}

Table S3. The built-in voltage (V_{bi}), relative dielectric constant (ε_r) and width of depletion region (W_d, measured at 0 V) of devices with and without HIES layer were determined.

Device	V_{bi} [V]	ε_r	W [nm]
control	0.57	3.92	234
with PFN-Br	0.65	2.69	234
with PFN-BArF$_4$	0.59	3.01	241

Table S4. The n, J_0, R_s, R_{sh}, A, B from simulated J_{dark}-V curves of control devices and devices with PFN-Br or PFN-BArF$_4$. The photosensitive layer is PTB7-Th:PC$_{71}$BM.

Device	n	J_0 [A cm^{-2}]	R_s [Ω cm^2]	R_{sh} [Ω cm^2]	A	B
control	1.91	3.45×10^{-10}	24	1.5×10^{5}	9.8×10^{-8}	4.0
PFN-Br	1.77	9.19×10^{-11}	41	3.4×10^{6}	4.4×10^{-8}	4.1
PFN-BArF$_4$	1.90	2.61×10^{-10}	42	6.3×10^{5}	3.3×10^{-8}	4.0

Table S5. The n, J_0, R_s, R_{sh}, A, B from simulated J_{dark}-V curves of control devices and devices with HIES. The photosensitive layer is PTB7-Th:Y-QC4F.

Device	n	J_0 [A cm^{-2}]	R_s [Ω cm^2]	R_{sh} [Ω cm^2]	A	B
control	3.6	1.7×10^{-7}	37	1.9×10^{4}	1.3×10^{-5}	5.6
with HIES	1.6	1.2×10^{-7}	51	1.7×10^{6}	7.7×10^{-6}	6.0

Table S6. The n, J_0, R_s, R_{sh}, A, B from simulated J_{dark}-V curves of control devices and devices with HIES. The photosensitive layer is PbS/JD40-BDD20.

Device	n	J_0 [A cm^{-2}]	R_s [Ω cm^2]	R_{sh} [Ω cm^2]	A	B
control	3.6	1.7×10^{-7}	37	1.9×10^{4}	1.3×10^{-5}	5.6
with HIES	1.6	1.2×10^{-7}	51	1.7×10^{6}	7.7×10^{-6}	6.0

Table S7. The figure of merit of the optimized SWIR PDs compared with reported solution-processed SWIR PDs and the commercial inorganic SWIR PDs.

Photosensitive layer	λ [nm]	EQE [%]	J_{dark} [A cm^{-2}]	D^* [Jones]	Ref.
PbS CQDs	1550	80.0	2.0×10^{-6}	8.0×10^{11}	[51]
PbS CQDs	1450	17.0	2.0×10^{-7}	1.0×10^{11}	[78]
PbS CQDs	1520	7.5	6.0×10^{-8}	2.9×10^{12}	[79]
PbS CQDs	1520	73.0	1.0×10^{-6}	1.6×10^{12}	[80]
PbS CQDs	1500	40.0	1.4×10^{-7}	1.4×10^{12}	[81]
PbS CQDs	1450	40.0	3.3×10^{-6}	9.5×10^{11}	[82]
PbS CQDs	1300	70.0	3.0×10^{-7}	4.1×10^{11}	[83]
PbS CQDs	1330	42.0	1.0×10^{-7}	3.9×10^{11}	[84]
PbS CQDs	1300	-	1.6×10^{-2}	1.8×10^{13}	[85]
Ag_2Te CQDs	1500<br>1350	8.3	2.0×10^{-6}	8.8×10^{11}<br>3.0×10^{12}	[20]
HgTe CQDs	1640	-	2.3×10^{-6}	3.9×10^{11}	[86]
PbS CQDs (hybrid)	1220	16.5	2.5×10^{-5}	2.0×10^{9}	[87]
PbS CQDs (hybrid)	1550	7.2	-	5.2×10^{11}	[88]
Organic	1400	10.5	-	8.2×10^{10}	[89]
Organic	1275<br>1600	7.8	-	2.0×10^{13}<br>2.0×10^{11}	[90]
Organic	1160<br>1500	13.4<br>0.4	3.2×10^{-10}	1.7×10^{12}<br>1.0×10^{11}	[50]
PbS CQDs/organic	1520<br>1550	57.7<br>53.0	6.7×10^{-8}	4.4×10^{12}<br>4.3×10^{12}	This work

Data are taken from refs. [20, 44, 45, 78-90]

References

1. Gong X, Tong M, Xia Y *et al.* High-detectivity polymer photodetectors with spectral response from 300 nm to 1450 nm. *Science* 2009; **325**: 1665–7.
2. García de Arquer FP, Talapin DV, Klimov VI *et al.* Semiconductor quantum dots: technological progress and future challenges. *Science* 2021; **373**: eaaz8541.
3. Bianconi S and Mohseni H. Recent advances in infrared imagers: toward thermodynamic and quantum limits of photon sensitivity. *Rep Prog Phys* 2020; **83**: 044101.
4. Geum D-M, Kim SK, Lee S *et al.* Monolithic 3D integration of InGaAs photodetectors on Si MOSFETs using sequential fabrication process. *IEEE Electron Device Lett* 2020; **41**: 433–6.
5. Piels M, Bauters JF, Davenport ML *et al.* Low-loss silicon nitride AWG demultiplexer heterogeneously integrated with hybrid III–V/silicon photodetectors. *J Light Technol* 2014; **32**: 817–23.
6. Lei W, Antoszewski J and Faraone L. Progress, challenges, and opportunities for HgCdTe infrared materials and detectors. *Appl Phys Rev* 2015; **2**: 041303.
7. Chow PCY and Someya T. Organic photodetectors for next-generation wearable electronics. *Adv Mater* 2020; **32**: 1902045.
8. Wang Y, Kubliitski J, Xing S *et al.* Narrowband organic photodetectors-towards miniaturized, spectroscopic sensing. *Mater Horiz* 2022; **9**: 220–51.
9. Fuentes-Hernandez C, Chou W-F, Khan TM *et al.* Large-area low-noise flexible organic photodiodes for detecting faint visible light. *Science* 2020; **370**: 698–701.
10. Liu M, Yazdani N, Yarema M *et al.* Colloidal quantum dot electronics. *Nat Electron* 2021; **4**: 548–58.
11. Manders JR, Lai T-H, An Y *et al.* Low-noise multispectral photodetectors made from all solution-processed inorganic semiconductors. *Adv Funct Mater* 2014; **24**: 7205–10.
12. Almeida G, Ubbink RF, Stam M *et al.* InP colloidal quantum dots for visible and near-infrared photonics. *Nat Rev Mater* 2023; **8**: 742–58.
13. Liu J, Liu P, Chen D *et al.* A near-infrared colloidal quantum dot imager with monolithically integrated readout circuitry. *Nat Electron* 2022; **5**: 443–51.
14. García De Arquer FP, Armin A, Meredith P *et al.* Solution-processed semiconductors for next-generation photodetectors. *Nat Rev Mater* 2017; **2**: 16100.
15. Li T, Hu G, Tao L *et al.* Sensitive photodetection below silicon bandgap using quinoid-capped organic semiconductors. *Sci Adv* 2023; **9**: eadf6152.
16. Li T, Hu G, Wu H *et al.* Highly sensitive water pollution monitoring using colloid-processed

organic photodetectors. *Nat Water* 2024; **2**: 577–88.

17. Fièque B, Martineau L, Sanson E *et al.* Infrared ROIC for very low flux and very low noise applications. In: *Proc SPIE 8176, Sensors, Systems, and Next-Generation Satellites **XV*** 2011; **8176**: 389-401.
18. Gielen S, Kaiser C, Verstraeten F *et al.* Intrinsic detectivity limits of organic near-infrared photodetectors. *Adv Mater* 2020; **32**: 2003818.
19. Simone G, Dyson MJ, Meskers SCJ *et al*. Organic photodetectors and their application in large area and flexible image sensors: the role of dark current. *Adv Funct Mater* 2020; **30**: 1904205.
20. Wang Y, Peng L, Schreier J *et al.* Silver telluride colloidal quantum dot infrared photodetectors and image sensors. *Nat Photon* 2024; **18**: 236–42.
21. Chen Y, Zheng Y, Wang J *et al.* Ultranarrow-bandgap small-molecule acceptor enables sensitive SWIR detection and dynamic upconversion imaging. *Sci Adv* 2024; **10**: eadm9631.
22. Xia Y, Zhang J, Guo T *et al.* High-Speed Flexible Near-Infrared Organic Photodetectors for Self-Powered Optical Integrated Sensing and Communications. *Adv Funct Mater* 2025; **35**: 2412813.
23. Bi P, Wang J, Chen Z *et al.* Weak Near-Infrared Light Visualization Enabled by Smart Multifunctional Optoelectronics. *Adv Mater* 2025; **37**: 2416785.
24. Kublitski J, Hofacker A, Boroujeni BK *et al.* Reverse dark current in organic photodetectors and the major role of traps as source of noise. *Nat Commun* 2021; **12**: 551.
25. Sandberg OJ, Kaiser C, Zeiske S *et al.* Mid-gap trap state-mediated dark current in organic photodiodes. *Nat Photon* 2023; **17**: 368–74.
26. Liu T, Jia Z, Song Y *et al.* Near Infrared Self-Powered Organic Photodetectors with a Record Responsivity Enabled by Low Trap Density. *Adv Funct Mater* 2023; **33**: 2301167.
27. Wang J, Deng S, Hu J *et al.* Alcohol-Soluble n-Type Polythiophenes as Cathode Interlayer in Organic Photodetectors for Hole Blocking. *Adv Funct Mater* 2024; **34**: 2312502.
28. Gao Y. Surface analytical studies of interfaces in organic semiconductor devices. *Mater Sci Eng R Rep* 2010; **68**: 39–87.
29. Robertson J and Wallace RM. High-K materials and metal gates for CMOS applications. *Mater Sci Eng R Rep* 2015; **88**: 1–41.
30. Hung LS and Chen CH. Recent progress of molecular organic electroluminescent materials and devices. *Mater Sci Eng R Rep* 2002; **39**: 143–222.
31. Paulsen BD, Tybrandt K, Stavrinidou E *et al*. Organic mixed ionic–electronic conductors. *Nat Mater* 2020; **19**: 13–26.

32. Kousseff CJ, Halaksa R, Parr ZS *et al.* Mixed ionic and electronic conduction in small-molecule semiconductors. *Chem Rev* 2022; **122**: 4397–419.
33. Tang H, Bai Y, Zhao H *et al.* Interface engineering for highly efficient organic solar cells. *Adv Mater* 2024; **36**: 2212236.
34. Page ZA, Liu Y, Duzhko VV *et al.* Fulleropyrrolidine interlayers: Tailoring electrodes to raise organic solar cell efficiency. *Science* 2014; **346**: 441–4.
35. Tang CG, Ang MCY, Choo K-K *et al.* Doped polymer semiconductors with ultrahigh and ultralow work functions for ohmic contacts. *Nature* 2016; **539**: 536–40.
36. Berggren M, Crispin X, Fabiano S *et al.* Ion electron–coupled functionality in materials and devices based on conjugated polymers. *Adv Mater* 2019; **31**: 1805813.
37. Tordera D, Kuik M, Rengert ZD *et al.* Operational mechanism of conjugated polyelectrolytes. *J Am Chem Soc* 2014; **136**: 8500–3.
38. Tang CG, Syafiqah MN, Koh Q-M *et al.* Multivalent anions as universal latent electron donors. *Nature* 2019; **573**: 519–25.
39. Yang R, Garcia A, Korystov D *et al.* Control of interchain contacts, solid-state fluorescence quantum yield, and charge transport of cationic conjugated polyelectrolytes by choice of anion. *J Am Chem Soc* 2006; **128**: 16532–9.
40. Meyer J, Hamwi S, Kröger M *et al.* Transition metal oxides for organic electronics: energetics, device physics and applications. *Adv Mater* 2012; **24**: 5408–27.
41. Onat BM, Huang W, Masaun N *et al.* Ultra-low dark current InGaAs technology for focal plane arrays for low-light level visible-shortwave infrared imaging. In: *Proc SPIE 6542, Infrared Technology and Applications XXXIII* 2007; **6542**: 233–41.
42. Meng L, Zhang Y, Wan X *et al.* Organic and solution-processed tandem solar cells with 17.3% efficiency. *Science* 2018; **361**: 1094–8.
43. Zhang Z, Wang W, Jiang Y *et al.* High-brightness all-polymer stretchable LED with charge-trapping dilution. *Nature* 2022; **603**: 624–30.
44. Chen Z, Hu Z, Wu Z *et al.* Counterion-tunable n-type conjugated polyelectrolytes for the interface engineering of efficient polymer solar cells. *J Mater Chem A* 2017; **5**: 19447–55.
45. Seo JH, Nguyen T-Q. Electronic Properties of Conjugated Polyelectrolyte Thin Films. *J Am Chem Soc* 2008; **130**: 10042–3.
46. Zheng Y, Zhao J, Liang H *et al.* Double-Dipole Induced by Incorporating Nitrogen-Bromine Hybrid Cathode Interlayers Leads to Suppressed Current Leakage and Enhanced Charge Extraction in Non-Fullerene Organic Solar Cells. *Adv Sci* 2023; **10**: 2302460.
47. Png R-Q, Ang MCY, Teo M-H *et al.* Madelung and Hubbard interactions in polaron band

model of doped organic semiconductors. *Nat Commun* 2016; **7**: 11948.

48. Yu X, Marks TJ and Facchetti A. Metal oxides for optoelectronic applications. *Nat Mater* 2016; **15**: 383–96.
49. Li W, Xu Y, Meng X *et al.* Visible to near-infrared photodetection based on ternary organic heterojunctions. *Adv Funct Mater* 2019; **29**: 1808948.
50. Zhang Y, Chen J, Yang J *et al.* Sensitive SWIR organic photodetectors with spectral response reaching 1.5 µm. *Adv Mater* 2024; **36**: 2406950.
51. Vafaie M, Fan JZ, Morteza Najarian A *et al.* Colloidal quantum dot photodetectors with 10-ns response time and 80% quantum efficiency at 1,550 nm. *Matter* 2021; **4**: 1042–53.
52. Matsushima T, Kinoshita Y, Murata H. Formation of Ohmic hole injection by inserting an ultrathin layer of molybdenum trioxide between indium tin oxide and organic hole-transporting layers. *Appl Phys Lett* 2007; **91**: 253504.
53. Chuang C-HM, Brown PR, Bulović V, Bawendi MG. Improved performance and stability in quantum dot solar cells through band alignment engineering. *Nat Mater* 2014; **13**: 796–801.
54. Ubrig N, Ponomarev E, Zultak J *et al.* Design of van der Waals interfaces for broad-spectrum optoelectronics. *Nat Mater* 2020; **19**: 299–304.
55. Yu H, Wang Y, Tong Q *et al*. Anomalous light cones and valley optical selection rules of interlayer excitons in twisted heterobilayers. *Phys Rev Lett* 2015; **115**: 187002.
56. Qiu Y, Liu C-S, Shi X *et al*. Momentum matching induced giant magnetoresistance in two-dimensional magnetic tunnel junctions. *Phys Chem Chem Phys* 2023; **25**: 25344–52.
57. Jiang Y, Li Y, Liu F, Wang W, Su W, Liu W *et al.* Suppressing electron-phonon coupling in organic photovoltaics for high-efficiency power conversion. *Nat Commun* 2023; **14**: 5079.
58. Ge Z, Qiao J, Li Y, Song J, Duan X, Fu Z *et al.* Regulating Electron-Phonon Coupling by Solid Additive for Efficient Organic Solar Cells. *Angew Chem Int Ed* 2025; **64**: e202413309.
59. Presselt M, Herrmann F, Hoppe H, Shokhovets S, Runge E, Gobsch G. Influence of Phonon Scattering on Exciton and Charge Diffusion in Polymer-Fullerene Solar Cells. *Adv Energy Mater* 2012; **2**: 999–1003.
60. Dettmann MA, Cavalcante LSR, Magdaleno CA, Moulé AJ. Catching the killer: dynamic disorder design rules for small-molecule organic semiconductors. *Adv Funct Mater* 2023; **33**: 2213370.
61. Do TT, Hong HS, Ha YE *et al.* Effect of polyelectrolyte electron collection layer counteranion on the properties of polymer solar cells. *ACS Appl Mater Interfaces* 2015; **7**: 3335–41.

62. Ma X, Bin H, van Gorkom BT *et al.* Identification of the origin of ultralow dark currents in organic photodiodes. *Adv Mater* 2023; **35**: 2209598.
63. Zeiske S, Sandberg OJ, Zarrabi N *et al.* Direct observation of trap-assisted recombination in organic photovoltaic devices. *Nat Commun* 2021; **12**: 3603.
64. Ni Z, Bao C, Liu Y *et al.* Resolving spatial and energetic distributions of trap states in metal halide perovskite solar cells. *Science* 2020; **367**: 1352–8.
65. Fang Y, Armin A, Meredith P *et al.* Accurate characterization of next-generation thin-film photodetectors. *Nat Photon* 2019; **13**: 1–4.
66. Yang Q, Fuchs F, Schmitz J, Pletschen W. Investigation of trap-assisted tunneling current in InAs/(GaIn)Sb superlattice long-wavelength photodiodes. *Appl Phys Lett* 2002; **81**:4757-9.
67. Wang Y, Hu H, Yuan M, Xia H, Zhang X, Liu J *et al.* Colloidal PbS Quantum Dot Photodiode Imager with Suppressed Dark Current. *ACS Appl Mater Interfaces* 2023; **15**: 58573–82.
68. Liu Y, Liu J, Deng C *et al.* Planar Cation Passivation on Colloidal Quantum Dots Enables High-Performance 0.35–1.8 μm Broadband TFT Imager. *Adv Mater* 2024; **36**: 2313811.
69. Hamamatsu. *Technical Note / Compound Opto-Semiconductor Photosensors*. https://www.hamamatsu.com/content/dam/hamamatsu-photonics/sites/documents/99_SALES_LIBRARY/ssd/compound_kird9004e.pdf.
70. Zhang J, Huang Q, Zhang K *et al.* Random copolymerization strategy for non-halogenated solvent-processed all-polymer solar cells with a high efficiency of over 17%. *Energy Environ Sci* 2022; **15**: 4561–71.
71. Park I, Kim C, Kim R *et al.* High performance shortwave infrared organic photodetectors adopting thiadiazole quinoxaline-based copolymers. *Adv Opt Mater* 2022; **10**: 2200747.
72. Huang F, Hou L, Wu H *et al.* High-efficiency, environment-friendly electroluminescent polymers with stable high work function metal as a cathode: green- and yellow-emitting conjugated polyfluorene polyelectrolytes and their neutral precursors. *J Am Chem Soc* 2004; **126**: 9845–53.
73. Hines MA and Scholes GD. Colloidal PbS nanocrystals with size-tunable near-infrared emission: observation of post-synthesis self-narrowing of the particle size distribution. *Adv Mater* 2003; **15**: 1844–9.
74. De Castro IA, Datta RS, Ou JZ, Castellanos-Gomez A, Sriram S, Daeneke T *et al.* Molybdenum oxides – from fundamentals to functionality. *Adv Mater* 2017; **29**: 1701619.
75. Greiner MT, Helander MG, Tang W-M, Wang Z-B, Qiu J, Lu Z-H. Universal energy-level

alignment of molecules on metal oxides. *Nat Mater* 2012; **11**: 76–81.

76. He Z, Zhong C, Su S, Xu M, Wu H, Cao Y. Enhanced power-conversion efficiency in polymer solar cells using an inverted device structure. *Nat Photon* 2012; **6**: 591–595.
77. Zheng Y, Zhao J, Liang H, Zhao Z, Kan Z. Double-dipole induced by incorporating nitrogen-bromine hybrid cathode interlayers leads to suppressed current leakage and enhanced charge extraction in non-fullerene organic solar cells. *Adv Sci* 2023; **10**: 2302460.
78. Clifford JP, Konstantatos G, Johnston KW *et al.* Fast, sensitive and spectrally tuneable colloidal-quantum-dot photodetectors. *Nat Nanotechnol* 2009; **4**: 40–4.
79. Klem EJD, Gregory C, Temple D *et al*. PbS colloidal quantum dot photodiodes for low-cost SWIR sensing. In: *Proc SPIE 9451*, *Infrared Technology and Applications XLI*, 2015; **9451**: 945104.
80. Biondi M, Choi M-J, Wang Z *et al.* Facet-oriented coupling enables fast and sensitive colloidal quantum dot photodetectors. *Adv Mater* 2021; **33**: 2101056.
81. Sliz R, Lejay M, Fan JZ *et al.* Stable colloidal quantum dot inks enable inkjet-printed high-sensitivity infrared photodetectors. *ACS Nano* 2019; **13**: 11988–95.
82. Pejović V, Lee J, Georgitzikis E *et al.* Thin-film photodetector optimization for high-performance short-wavelength infrared imaging. *IEEE Electron Device Lett* 2021; **42**: 1196–9.
83. Liu Y, Gao Y, Yang Q *et al.* Breaking the size limitation of directly-synthesized PbS quantum dot inks toward efficient short-wavelength infrared optoelectronic applications. *Angew Chem Int Ed* 2023; **62**: e202300396.
84. Deng Y-H, Pang C, Kheradmand E *et al.* Short-wave infrared colloidal QD photodetector with nanosecond response times enabled by ultrathin absorber layers. *Adv Mater* 2024; **36**: 2402002.
85. Konstantatos G, Howard I, Fischer A *et al.* Ultrasensitive solution-cast quantum dot photodetectors. *Nature* 2006; **442**: 180–3.
86. Yang J, Hu H, Lv Y *et al.* Ligand-engineered HgTe colloidal quantum dot solids for infrared photodetectors. *Nano Lett* 2022; **22**: 3465–72.
87. Rauch T, Böberl M, Tedde SF *et al.* Near-infrared imaging with quantum-dot-sensitized organic photodiodes. *Nat Photon* 2009; **3**: 332–6.
88. Ding N, Xu W, Liu H *et al.* Highly DUV to NIR-II responsive broadband quantum dots heterojunction photodetectors by integrating quantum cutting luminescent concentrators. *Light Sci Appl* 2024; **13**: 289.
89. Zimmerman JD, Yu EK, Diev VV *et al.* Use of additives in porphyrin-tape/C60 near-

infrared photodetectors. *Org Electron* 2011; **12**: 869–73.

90. Han J, Yang D, Ma D *et al*. Low-bandgap polymers for high-performance photodiodes with maximal EQE near 1200 nm and broad spectral response from 300 to 1700 nm. *Adv Opt Mater* 2018; **6**: 1800038.
