# Supplementary material for: Hybrid ionic–electronic semiconductors for interface engineering of ultra-low-dark-current solution-processed SWIR photodetectors
Source: Natl Sci Rev. 2025 Nov 26;13(2):nwaf531. doi: 10.1093/nsr/nwaf531 (PMC12831028; doi:10.1093/nsr/nwaf531)
Supplement: nwaf531_Supplemental_Files [file nwaf531_supplemental_files.zip › Teaser text.docx]

Teaser text

By leveraging hybrid ionic–electronic semiconductors, a universally applicable strategy breaks the performance bottleneck of solution-processed SWIR photodetectors and unlocks high-performance, low-cost SWIR imaging.
